# Alternate approach to stroke phenotyping identifies a genetic risk locus for small vessel stroke

**DOI:** 10.1038/s41431-020-0580-5

**Published:** 2020-02-11

**Authors:** Joanna von Berg, Sander W. van der Laan, Patrick F. McArdle, Rainer Malik, Steven J. Kittner, Braxton D. Mitchell, Bradford B. Worrall, Jeroen de Ridder, Sara L. Pulit

**Affiliations:** 1 Genetics, Center for Molecular Medicine, University Medical Centre Utrecht, Utrecht, The Netherlands; 2 Central Diagnostics Laboratory, Division Laboratories, Pharmacy, and Biomedical Genetics, University Medical Center Utrecht, Utrecht, The Netherlands; 3 Division of Endocrinology, Diabetes, and Nutrition, Department of Medicine, University of Maryland School of Medicine, Baltimore, MD USA; 4 Institute for Stroke and Dementia Research (ISD), University Hospital, LMU Munich, Munich, Germany; 5 Department of Neurology, Veterans Affairs Maryland Healthcare System and University of Maryland School of Medicine, Baltimore, MD USA; 6 Departments of Neurology and Public Health Sciences, University of Virginia, Charlottesville, VA USA; 7 Oncode Institute, Utrecht, The Netherlands; 8 Big Data Institute, Li Ka Shing Center for Health Information and Discovery, Oxford University, Oxford, UK; 9 Program in Medical and Population Genetics, Broad Institute, Boston, MA USA

**Keywords:** Stroke, Genome-wide association studies, Quantitative trait, Genetic variation, Genetics research

## Abstract

Ischemic stroke (IS), caused by obstruction of cerebral blood flow, is one of the leading causes of death. While neurologists agree on delineation of IS into three subtypes (cardioembolic stroke (CES), large artery stroke (LAS), and small vessel stroke (SVS)), several subtyping systems exist. The most commonly used systems are TOAST (Trial of Org 10172 in Acute Stroke Treatment) and CCS (Causative Classification System for Stroke), but agreement is only moderate. We have compared two approaches to combining the existing subtyping systems for a phenotype suited for a genome-wide association study (GWAS). We used the NINDS Stroke Genetics Network dataset (SiGN, 11,477 cases with CCS and TOAST subtypes and 28,026 controls). We defined two new phenotypes: the intersect, for which an individual must be assigned the same subtype by CCS and TOAST; and the union, for which an individual must be assigned a subtype by either CCS or TOAST. The union yields the largest sample size while the intersect yields a phenotype with less potential misclassification. We performed GWAS for all subtypes, using the original subtyping systems, the intersect, and the union as phenotypes. In each subtype, heritability was higher for the intersect compared with the other phenotypes. We observed stronger effects at known IS variants with the intersect compared with the other phenotypes. With the intersect, we identify rs10029218:G>A as an associated variant with SVS. We conclude that this approach increases the likelihood to detect genetic associations in ischemic stroke.

## Introduction

Stroke is one of the primary causes of death worldwide and causes approximately one in every 20 deaths in the United States [1]. Eighty-seven percent of all strokes are ischemic, caused by a blockage of blood flow to the brain [1]. Ischemic stroke (IS) tends to affect those older than 65 years old and has several known risk factors, including type 2 diabetes, hypertension, and smoking. However, the affected population is extremely heterogeneous in terms of age, sex, ancestral background, and socioeconomic status.

ISs themselves are also heterogeneous in terms of clinical features and presumed mechanism. The majority of IS are typically grouped into three subtypes: cardioembolic stroke (CES), frequently occurring in people with atrial fibrillation; large artery stroke (LAS), caused by eroded or ruptured atherosclerotic plaques in arteries; and small vessel stroke (SVS), caused by a blockage of one of the small vessels in the brain. These subtypes also seem to be genetically distinct: genome-wide association studies (GWAS) in ISs have identified single-nucleotide polymorphisms (SNPs) that primarily associate with a specific IS subtype [2]. To date, GWAS have identified 20 loci associated with IS, of which nine appear to be specific to an IS subtype [2]. Furthermore, the subtypes also have varying SNP-based heritabilities (estimated at 16%, 12%, and 18% for CES, LAS, and SVS respectively [3]), indicating that the phenotypic variation captured by genetic factors varies across the subtypes. Improved genetic discovery can help further elucidate the underlying biology of IS as well as potentially help identify genetically high-risk patients who could be candidates for earlier clinical interventions.

While neurologists and researchers agree on the delineation of IS into these three primary categories (CES, LAS, and SVS), multiple subtyping systems are currently used to assign a subtype to an IS patient. The most commonly used approach is a questionnaire based on clinical knowledge that was originally developed for the Trial of Org 10172 in Acute Stroke Treatment (TOAST) [4]. TOAST was designed for implementation in the clinic and has also been used as the subtyping system in the majority of stroke GWAS. More recently, researchers have developed a second subtyping system: the Causative Classification System for Stroke (CCS) [5], a decision model based on clinical knowledge that also incorporates imaging data. There are two outputs of CCS: CCS Causative (CCSc), which assigns one subtype to each patient based on the presumed cause of the stroke; and CCS phenotypic (CCSp), which allows for multiple subtype assignments and incorporates the confidence of the assignment. Previous work indicates that TOAST and CCS have moderate, but not high, concordance in assigning subtypes in patients: agreement is lowest in SVS (κ = 0.56) and highest in LAS (κ = 0.71) [6]. Notably, both subtyping systems still place more than one third of all samples into a heterogeneous ‘undetermined’ category [6].

**Fig. 1:**
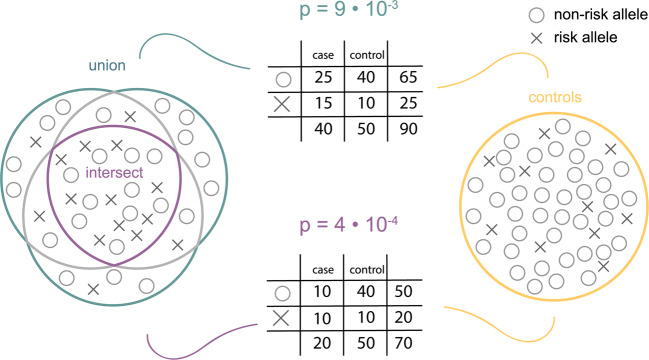
Hypothesized benefit of using the intersect, at a SNP associated with ischemic stroke. Circles indicate the non-risk allele, and crosses the risk allele. Using a chi-square test (visualized with contingency tables), the measured effect is stronger with a group of cases that is more homogeneous but smaller (intersect, purple) than with a group of cases that is less strictly defined but is larger (union, teal).

Determining a patient’s subtype is difficult and prone to misclassification [7], but critical to genetic discovery in IS, as demonstrated by the prevalence of subtype-specific association signals. If a group of cases is comprised of phenotypically heterogeneous samples with different underlying genetic risk, power to detect a statistically significant association at a truly associated SNP is reduced (Fig. 1). In contrast, a case definition that captures a more phenotypically homogenous group of cases would improve the chances of detecting genetic variants that associate with disease. Therefore, we used the TOAST, CCSc, and CCSp subtype assignments to define two new phenotypes per subtype: the intersect, for which an individual must be assigned the same subtype across all three subtyping systems; and the union, for which an individual must be assigned that subtype by at least one of the subtyping systems. Analyzing the union potentially improves power for locus discovery due to its larger sample size, but at the cost of more potential misclassification. In contrast, analyzing the intersect may improve power for genetic discovery by generating a phenotype that is less prone to misclassification, despite a smaller sample size.

Here, we perform GWAS with the union and intersect phenotypes for each primary IS subtype to investigate whether these newly defined phenotypes indeed improve our ability to detect genetic risk factors for IS. We find heritability estimates to be highest in the intersect phenotype for all subtypes. We also find stronger effects at known associations for the intersect compared with the union, and we validate a previously suspected association in SVS through GWAS of the intersect phenotype.

## Results

### Genome-wide association study data processing

To investigate how redefining stroke phenotypes improves our ability to detect SNPs associated with IS, we employed the Stroke Genetics Network (SiGN) dataset. Data processing of the SiGN dataset, including quality control and imputation, have been described in detail elsewhere [8]. Briefly, the dataset includes 13,930 IS cases and 28,026 controls of primarily European descent. Cases and controls were genotyped separately (with the exception of a small number of cohorts) and on various Illumina arrays and then merged together into case-control groups matched for genotyping array and sample ancestry (via principal component analysis). For the cases, phenotype definitions based on one or more of the CCSc, CCSp, and TOAST subtyping systems are available (Table 1). The total number of cases with subtype information available is 11,477.

**Table 1:** Case counts for the different phenotype definitions in the three subtypes.

	CES	LAS	SVS	Undetermined (‘other’ for CCSp)	Total
C (CCSc)	3,000	1,565	2,262	4,574	11,401
P (CCSp)	3,608	2,449	2,419	718	9,194
T (TOAST)	3,333	2,318	2,631	3,479	11,761
I (intersect)	2,219	1,328	1,548	Not tested	5,095
U (union)	4,502	3,495	3,480	Not tested	11,477
S (symmetric difference)	2,283	2,167	1,932	Not tested	6,382

The control group is always the same group of 28,026 individuals.

We began our analyses by running GWAS for all phenotype definitions, including our intersect and union definitions, in all subtypes. We ran all GWAS using a linear mixed model (LMM) implemented in BOLT-LMM (Fig. S1) [9], adjusting for sex. To take into account any residual population stratification and other batch effects, we included the first ten principal components as covariates in these analyses (Table S2).

Because the intersect by definition is contained in the union, we ran one additional GWAS for each subtype to enable a statistically independent comparison of the intersect with the symmetric difference (the union minus the intersect). This study focuses on the balance in statistical power between a high sample size (union) and more strictly defined phenotype (intersect). Therefore, this sensitivity analysis was only done for the comparison between the two most extreme case definitions (union and intersect), and not for the comparisons between the other phenotypes, where overlap in samples also exists.

### Genetic variance in a strictly defined case group explains a higher proportion of phenotypic variance

**Fig. 2:**
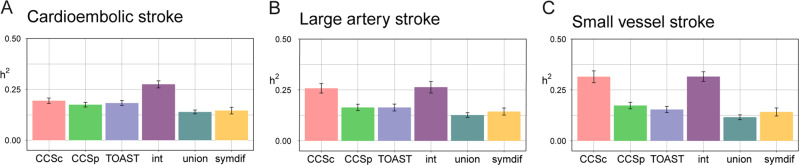
Intersect is the most heritable phenotype. Heritabilities on the liability scale for the six case definitions. Bars indicate the standard error. Note that intersect has a relatively high standard error, due to its lower sample size. **a** In cardioembolic stroke, intersect is significantly more heritable than all other phenotype definitions (*p* values for the difference between intersect and all others 3.6e−03 or lower). **b** In large artery stroke and **c** small vessel stroke, intersect is significantly more heritable than all other phenotype definitions except CCSc (*p* values for the difference between intersect and all others except CCSc, 2.7e−03 or lower in LAS, 6.1e−07 or lower in SVS). *P* values for heritability differences determined by *t*-test (see Table S5). See Table S4 for numerical values of heritabilities and standard errors. int intersect, symdif symmetric difference.

To estimate how much of the variation in a particular phenotype can be explained by genetic variation, we calculated the heritability (*h*^*2*^) of each phenotype using BOLT-REML, assuming an additive model of effect sizes overall SNPs. We estimated heritability in each of the available phenotypes: the subtypes as defined by TOAST, CCSc, CCSp, the union, and the intersect. We found that the intersect yields a higher *h*^*2*^ than the union in all IS subtypes (Fig. 2 and Table S3). For instance, in CES, *h*^*2*^ of union is 0.139 ± 0.009 and *h*^*2*^ of intersect is 0.275 ± 0.017. We additionally found that the second highest heritability estimate in large artery and SVS was in CCSc (*h*^*2*^-LAS = 0.258 ± 0.023 and *h*^*2*^-SVS = 0.315 ± 0.029), which assigns only one subtype to each case. The heritabilities for CCSc, CCSp, and TOAST were not significantly different from one another in CES (Table S4), indicating that each original subtyping system is capturing approximately the same proportion of genetic risk.

### Different phenotype definitions represent genetically distinct phenotypes

**Fig. 3:**
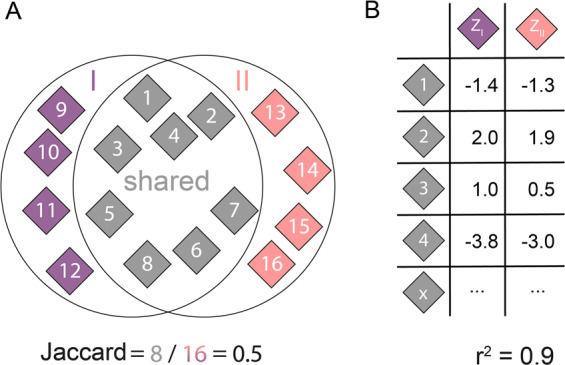
Graphical explanation of overlap analysis. **a** At a certain absolute *z*-score threshold *Z*, all SNPs that have a z-score lower than −*Z* or higher than +*Z* in GWAS I are determined (SNPs 1–8 and 9–12). Next, all SNPs that have a z-score lower than −*Z* or higher than +*Z* in GWAS II are determined (SNPs 1–8 and 13–16). The number of shared significant SNPs is divided by the union of significant SNPs to calculate the Jaccard index. **b** We also calculate the Pearson correlation of the *z*-scores of the shared SNPs.

While heritability gives an estimation of how much variation in a phenotype can be attributed to genetic factors, it does not show how different two phenotypes are from one another (i.e., two phenotypes can have the same heritability and yet be genetically distinct from each other). We therefore evaluated the overlap in significant SNPs for all pairwise combinations of phenotypes for which we performed a GWAS, where high proportions of shared SNPs between two phenotypes indicate genetic similarity. At multiple significance cutoffs, we assessed overlap of significant SNP sets using two complementary similarity measures: the Jaccard index, which measures the ratio of overlapping SNPs (those are significant in both analyses) in the total set of SNPs that are significant in either analysis; and the Pearson correlation of the *z*-scores of the overlapping SNPs in both analyses (Fig. 3). Significance is defined here as an absolute *z*-score that is higher than the selected *z*-score threshold (where SNPs can have an effect size < −Z or > + Z). A high Jaccard index indicates that two phenotypes share many of their associated SNPs, while a low Jaccard index means that the phenotypes have distinct genetic architecture. Correlation pertains only to the shared SNPs and indicates if they have similar directionality and magnitude of effect in both analyses.

In order to assess the results of the overlap analyses and their meaning with respect to the IS phenotypes, we also performed these analyses between the phenotype definitions and an unrelated GWAS of educational attainment to obtain a null reference (Fig. S2).

**Fig. 4:**
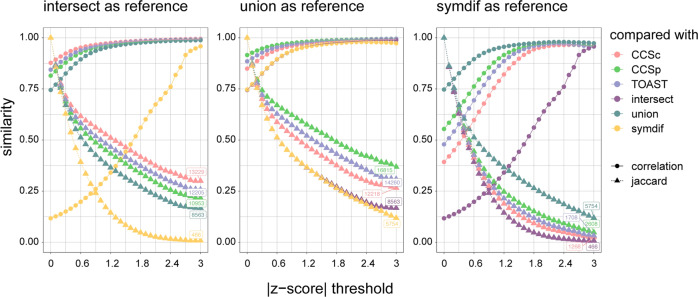
Different phenotype definitions capture different genetic risk factors. Overlap analysis in cardioembolic stroke. Similarity on the *y*-axis denotes either correlation (circles) or Jaccard index (triangles). The absolute *z*-score threshold is plotted on the *x*-axis. Numbers indicate the number of shared SNPs at *Z* = 3. **a** Pairwise comparisons with intersect, **b** pairwise comparisons with union, **c** pairwise comparisons with symmetric difference.

In CES (Fig. 4, first panel), the Jaccard index for all combinations with intersect decreases with more extreme *z*-scores to *J* ≈ 0.2–0.3 while the correlation increases quickly to approach *r*^2^ = 1 at *Z* ≈ 2.5. These results indicate that a relatively small group of SNPs is significant in both analyses with correlating *z*-scores that get increasingly smaller and stronger correlating. These findings indicate that the stricter the significance threshold is, the fewer shared SNPs there are between any two phenotypes, but that those shared SNPs have more concordant effect sizes. In LAS (Fig. S3) and SVS (Fig. S3) the trend is similar, albeit with lower Jaccard indices and correlations, suggesting that there is a set of associated SNPs for each subtype that is found by all phenotype definitions. In all subtypes, when compared with symmetric difference, the intersect is the most genetically distinct phenotype. This confirms that if we combine symmetric difference and intersect, as in the union, we increase phenotypic heterogeneity and thereby decrease the likelihood of detecting a genome-wide significant signal.

Figure 4 shows pairwise comparisons only; to investigate if there is one group of SNPs that is significant in all analyses, we also calculated overall Jaccard index: the size of the intersect of SNPs that are significant in all five phenotypes (excluding symmetric difference, which we use for sensitivity testing only), divided by the size of their union. The overall Jaccard index (Fig. S4) confirms what was suggested by the pairwise overlap analyses: there is a small set of SNPs that is shared across all phenotype definitions, albeit slightly smaller than the pairwise overlapping sets. The Jaccard index is relatively low at higher significance thresholds, indicating that there is also a substantial set of SNPs that is unique to each phenotype definition. Thus, we do find different associated SNPs to IS subtypes depending on how exactly the subtype status is defined, but there are some concordant SNPs that are found by all case definitions, regardless of sample size or phenotype homogeneity.

### Intersect shows the largest effect at previously known associations

**Fig. 5:**
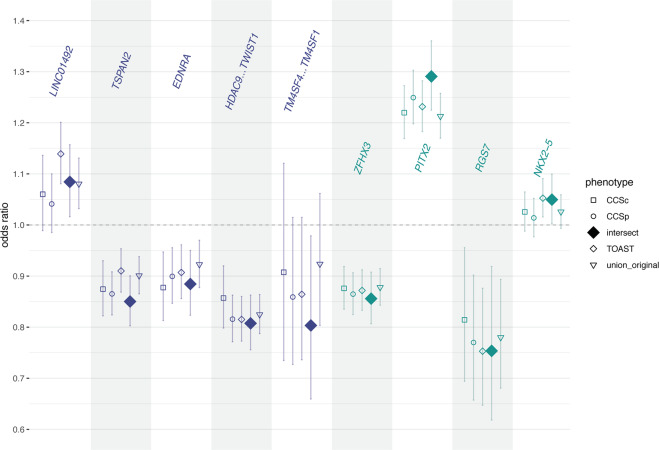
Intersect most often shows the strongest effect at previously identified subtype-specific associations. Odds ratios for the five LAS-associated SNPs (in purple) and the four CES-associated SNPs (in teal) in the five phenotype definitions. The dotted line indicates an OR of 1 (no effect). Error bars indicate the 95% confidence interval. Intersect show the strongest effect at five of the nine SNPs (binomial *p* = 0.0196).

A recent GWAS (MEGASTROKE) in 67,162 TOAST-subtyped cases and 454,450 controls identified 32 loci (22 novel) associated to stroke (either IS or intracerebral hemorrhage) and its subtypes [2]. Four of the 32 loci associate to CES, five to LAS, and none to SVS. We investigated the potential to find stroke-associated loci in our redefined phenotypes, with a sample that is four to seven times smaller than MEGASTROKE. To this end, we compared the odds ratios (ORs) in the intersect with the ORs in the other phenotype definitions, at the nine known subtype-specific SNPs (Fig. 5). In five of the nine known SNPs, the intersect shows the most extreme OR (binomial *p* = 0.0196) out of the five phenotype definitions.

Besides comparing the ORs at subtype-specific signals, we also compared ORs at all stroke-associated loci (including any stroke, any IS, CES, and LAS), see Fig. S5. We found that intersect shows the strongest OR 30 times out of 96, (binomial *p* = 0.010), indicating that ORs derived from the intersect phenotype are indeed stronger than the ORs in the other phenotypes more often than expected by chance.

### A stricter phenotype definition finds a new associated locus to small vessel stroke

Our analyses revealed five new loci (two for SVS and three for CES, Table 2 and Fig. S6) at a *p* value threshold of 5 × 10^−8^. We validated these signals using data from MEGASTROKE (based on the summary statistics of MEGASTROKE with the SiGN cohort removed, to ensure sample independence). We corrected the common *p* value threshold of 0.05 for a lookup of multiple SNPs per subtype. For SVS the corrected threshold is *α* = 0.0125 (two SNPs) and for CES *α* = 0.00833 (three SNPs).

**Table 2:** Summary statistics for the new genome-wide significant SNPs.

Locus	SNP	Chr	A1	A2	Analysis	*P* value	Freq1	OR	Beta	SE
CAMK2D	rs10029218	4	A	G	SVS-intersect	1.20E−08	0.12	1.27	0.02	0.00
					SVS-rep-EUR	2.46E−02	0.11	1.11	0.10	0.05
					SVS-rep-TRANS	**5.98E−03**	0.13	1.12	0.11	0.04
SH2B3-BRAP-ALDH2	rs11065979	12	T	C	SVS-CCSp	9.40E−09	0.42	1.13	0.01	0.00
					SVS-rep-EUR	**7.62E−03**	0.43	1.08	0.08	0.03
					SVS-rep-TRANS	**9.29E−03**	0.41	1.08	0.07	0.03
PFH20	rs11697087	20	A	G	CES-intersect	3.20E−09	0.09	1.26	0.02	0.00
					CES-rep-EUR	1.55E−02	0.09	1.10	0.10	0.04
					CES-rep-TRANS	4.76E−02	0.09	1.07	0.07	0.03
5:114799266	rs2169955	5	T	C	CES-CCSc	3.90E−08	0.57	0.90	−0.01	0.00
					CES-rep-EUR	1.48E−02	0.56	0.95	−0.05	0.02
					CES-rep-TRANS	2.22E−02	0.56	0.96	−0.04	0.02
GNAO1	rs3790099	16	C	G	CES-CCSp	4.90E−08	0.85	0.87	−0.02	0.00
					CES-rep-EUR	**2.97E−04**	0.84	0.89	−0.11	0.03
					CES-rep-TRANS	1.10E−02	0.77	0.94	−0.07	0.03

Per locus, the SiGN GWAS is in the first row, in the format ‘subtype–phenotype’. In the other rows, results in MEGASTROKE are shown with ‘subtype-rep-EUR’ for the Europeans-only analysis, and with ‘subtype-rep-TRANS’ for the trans-ancestry analysis. NB, Beta, and SE of SiGN GWAS and MEGASTROKE GWAS are not comparable since they come from linear and logistic regression, respectively. The ORs are comparable. We did a Bonferroni correction: for SVS, *α* = 0.0125 and for CES, *α* = 0.00625. Replication *p* values below the threshold are indicated in bold. Two SNPs (rs2169955:C>T and rs62379973:C>G, in CES-CCSc) that are relatively close (260 kb) on chromosome 5 were in two different clumps, even though they are in LD (*r*2 = 0.52, D′ = 0.87, in a CEU population [31]) because the distance is just above the threshold (250 kb). Because they are in LD, and just a little farther apart than 250 kb, they were considered to be from the same locus and only the strongest association was kept (rs2169955:C>T).

*A*1 allele 1, *A*2 allele 2, *Freq*1 frequency of allele 1, *OR* odds ratio, *Beta* coefficient, *SE* standard error.

We identified one variant (rs10029218:G>A, NC_000004.11:g.114401929G>A) in an intron of *CAMK2D* as associated to SVS in the intersect analysis. This variant replicated (*p* = 5.98 × 10^−3^) in the trans-ancestry analysis of MEGASTROKE. The other SVS associated variant (rs11065979:C>T, NC_000012.11:g.112059557C>T) in the *SH2B3*-*BRAP*-*ALDH2* locus was found in the CCSp analysis, and replicated in both the trans-ancestry analysis (*p* = 9.29 × 10^−3^) and the European analysis (*p* = 7.26 × 10^−3^). Of the three potential CES loci, we identified only one variant in the CCSp analysis (rs3790099:C>G, NC_000016.9:g.56340223C>G), an intronic variant in the *GNAO1* gene that replicated in the Europeans-only analysis of MEGASTROKE (*p* = 2.97 × 10^−4^). In a meta-analysis of (a) the MEGASTROKE GWAS without the SiGN cohort and (b) the SiGN GWAS, we found consistent direction of effect for these three SNPs in both studies and a more significant *p* value in the meta-analysis compared with either SiGN or MEGASTROKE alone (Table S5).

Previously, one other locus was reported to associate solely with SVS (16q24 [10]). Here, by applying an alternate phenotyping approach, we identify 4p12 as an additional SVS locus. In general, despite the low sample size as compared with MEGASTROKE, we find stronger associations in the intersect GWAS compared with the other phenotypes.

## Discussion

To help uncover genetic associations with IS that as yet have gone undetected, we defined new IS phenotypes based on three existing subtyping systems (CCSc, CCSp, and TOAST). Specifically, we studied the intersect and union of these subtyping systems, for all IS subtypes. The intersect results in a smaller number of available cases but potentially results in less misclassification due to agreement between subtyping systems. The union is potentially more heterogeneous, but results in a larger available group of cases. We find that the largest proportion of phenotypic variance explained by SNPs is in the intersect phenotype. Further, our overlap analyses show that, for each subtype, the phenotype definitions each have a unique set of significantly associated SNPs, but that there is also a small set of SNPs that is shared among all definitions, with concordant direction of effect and similar trend in magnitude of effect. We also show that the cases that are in the union but not in the intersect, are genetically distinct from the intersect-cases, implying that the union is a combination of phenotypically heterogeneous cases. With an effective sample size that is four to seven times as small as in MEGASTROKE, we find stronger associations (i.e., higher ORs and lower *p* values) at known loci by using the intersect (compared with the other phenotype definitions studied here). This indicates that the intersect yields more net power to detect associations due to its stricter definition, despite its lower sample size, and is thus better suited as a phenotype in GWAS.

We identify a previously subthreshold association with an SNP in an intron of the *CAMK2D* locus in SVS by using the intersect, further demonstrating the utility of this phenotype in GWAS. *CAMK2D* expresses a calcium/calmodulin-dependent protein kinase [11]; out of all tissues tested in GTEx, the two tissues with the highest expression are both in brain [12]. Further fine mapping in this region may give more insight into the biological mechanisms that contribute to stroke. In addition, we find the *SH2B3-BRAP-ALDH2* locus to be associated with SVS. rs11065979:C>T is an eQTL of *ALDH2* (aldehyde dehydrogenase 2) [12]. ALDH2 is involved in ethanol metabolism; it converts one of the products, ethanal, into acetic acid. The allele that is associated with higher expression of this enzyme is associated with lower incidence of SVS. *ALDH2* is mainly expressed in liver, but it is also expressed in brain [12]. Previous work has shown an association between higher expression of *ALDH2* and lower incidence of stroke in rats [13]*. SH2B3* and *BRAP* are minimally expressed in brain, compared with the other tissues [12]. We also show an association between an intronic SNP in *GNAO1* and CES. Little is known about the function of GNAO1, though it is expressed in brain and some data suggests that defects in the protein are associated with brain abnormalities [14]. Overall, the causal genes at these loci remain uncertain and further analyses within these loci are warranted.

In this study, we focused on the statistical comparison of CCS and TOAST, their union and their intersect for use in GWAS. It seems plausible to us that CCS and TOAST identify biologically different stroke cases. After all, they use different information to subtype a stroke patient and their agreement is moderate. The intersect might then be composed of those patients that harbor both the features that CCS deems important, as well as the features that TOAST deems important. This would result in a more homogeneous case group in the intersect, and thus increase the statistical power to detect associations. With the data we have presented here, we cannot explain what causes the differences in subtyping methods. Further studies aimed at answering this question might elucidate more of IS biology.

Phenotype definition is an oft-encountered issue in complex trait genetics, as diagnosing and subtyping methodologies can vary and even be contentious within disease areas. Further, phenotype labels are often broad definitions for cases that can be highly heterogeneous when their underlying genetic risk is examined. For example, most psychiatric diseases are also complex and phenotypically heterogeneous, lacking clear and robust diagnostic criteria. In an editorial, the Cross-Disorder Phenotype Group of the Psychiatric GWAS Consortium states: “We anticipate that genetic findings will not map cleanly onto current diagnostic categories and that genetic associations may point to more useful and valid nosological entities” [15]. Our findings here further support this statement, showing that while the original subtyping systems might be useful for diagnosing individual patients, alternative phenotyping approaches and criteria are needed for future genetic studies aimed at unraveling the underlying biology of disease.

## Methods

### The SiGN dataset

The SiGN Consortium composed a dataset consisting of 14,549 IS cases [16]. The control group consists primarily of publicly available controls drawn from three large multiancestry cohorts. Descriptions of the contributing case and control cohorts have been published previously [8]. Cases and controls were genotyped on a variety of Illumina arrays, and nearly all cases (~90%) were subtyped using both TOAST [4] and CCS [17]. All newly genotyped cases for the latest GWAS are available on dbGAP (accession number phs000615.v1.p1). A previous genome-wide association study was done on the separate TOAST and CCS subtypes [8]. In this work, we use the same 28,026 controls from this previous GWAS, as well as the 11,477 IS cases of European and African descent with subtype information available. A third group of cases and controls, primarily comprised of individuals who identify as Hispanic and residing in the United States, has been excluded due to data sharing restrictions. All data processing has been previously described [8]. All genotyping data are annoted on human genome build hg19.

### Genome-wide association studies in BOLT-LMM

We ran all GWAS in BOLT-LMM [9], which implements an LMM. BOLT-LMM implements a Leave-One-Chromosome-Out scheme, so that the genetic relationship matrix (GRM) is built on all chromosomes except the chromosome of the variant being tested. Linear mixed models have been demonstrated to improve power in GWAS while correcting for structure in the data [18]. In addition to the GRM, we included the first ten principal components as fixed effects. We used the following approximation to convert the effect estimates from BOLT-LMM (on the observed scale) to effect estimates on the liability scale: $\log \left( {OR} \right) = \beta /(\mu \times (1 - \mu ))$ where ${\mu}$ is the case fraction [19]. For each subtype, the intersect, union, and symmetric difference of the original subtyping systems were used as phenotypes in separate GWAS. The original subtyping systems were also used as a phenotype in three additional GWAS per subtype to serve as a point of reference. All IS cases that do not belong to the case definition under study were left out of the analysis. The same group of controls is used in all analyses. Association testing was done on all imputed SNPs with a minimum minor allele frequency of 1%. See Table S8 in [20] for simulations of type 1 error inflation of BOLT-LMM in datasets with unbalanced case-control ratios. In the GWAS discussed here, case fractions range from 0.05 to 0.14 which means that at variants with MAF > 1%, there is no significant inflation of type 1 error rates. Those SNPs that show a large frequency difference (>15%) across the populations in 1000 Genomes were removed (see the “Methods” in [8] for details on how this list of SNPs was compiled). See Fig. S1 for QQ-plots (stratified by imputation quality (INFO-score) and by minor allele frequency) and Manhattan-plots. The genomic inflation factor (lambda) varies between 1.0 and 1.1 for CES and LAS, and between 1.0 and 1.2 for SVS. We observed a relatively high inflation factor of 1.2 in only the imputed SNPs with a minor allele frequency lower than 5%. Therefore, summary statistics for these SNPs were removed from downstream analyses. Summary statistics for all GWAS can be found on Zenodo: 10.5281/zenodo.3514726.

### Heritability estimation in BOLT-REML

To estimate the heritability of the six phenotype definitions for each subtype, we used BOLT-REML [21]. BOLT-REML calculates heritability from the SNPs included in the GRM, and these SNPs must be genotyped (and not imputation dosages). We therefore based our estimates on only genotyped SNPs. Furthermore, we excluded the MHC on chromosome 6, and chromosomal inversions on chromosomes 8 and 17 using PLINK 1.9 [22]. See Table S8 for more information. We filtered on various quality control measures, by passing the following flags to PLINK: –mind 0.05 –maf 0.10 –geno 0.01 –hwe 0.001. In addition, we pruned SNPs at an LD (*r*^2^) threshold of 0.2 (–indep-pairwise 100 50 0.2). We used the first ten principal components and sex (determined by presence of XX or XY chromosomes) as covariates. To convert the heritabilities from the observed scale (as if the binary data, coded as 0–1, were continuous) to the liability scale (converting the heritabilities of the observed binary trait to the heritabilities of the underlying, unobserved, continuous liability of the trait), Dempster et al. derived a formula that takes into account the prevalence of the disease in the population [23]. In the case of ascertained case-control traits, where the population prevalence is not equal to the study prevalence, this has to be taken into account as well [24]:

$$\hat h_l^2 = \frac{{K^2(1 - K)^2}}{{P(1 - P)\varphi (t)^2}}\hat h_o^2,$$

where $\hat h_l^2$ is the heritability on the liability scale, *K* is the population prevalence, *P* is the study prevalence, *t* is the liability threshold, and $\hat h_o^2$ is the heritability on the observed scale. To test for significant difference between the estimated heritabilities, we performed an independent *t*-test.

### Overlap analysis

We first calculated *z*-scores using the following formula: *z* = beta/se, where beta is the effect size of the SNP and se is the standard error of the beta estimate. The *z*-scores thus have unit standard error, but we did not standardize them to zero mean (as is the conventional method for calculating *z*-scores) to maintain the original direction of effect. To assess overlap between two GWAS, we calculated the Jaccard index [25], which is the ratio of (a) the number of SNPs significant in both analyses, to (b) the number of SNPs significant in either analysis (i.e., the size of the intersect divided by the size of the union of the sets of significant SNPs). The index is a number between 0 and 1: it is 0 if the two sets of significant SNPs do not have any SNPs in common, and it is 1 if the two sets of significant SNPs completely overlap. We additionally calculated, within the set of SNPs that are significant in both analyses, the Pearson’s correlation of the *z*-scores in the two GWAS to check the concordance of direction and size of effect in the two analyses being compared. Significance was defined as a *z*-score that is more extreme than an absolute *z*-score threshold *z* (varied from 0 until 3, in increments of 0.1). At the most extreme *z*-score threshold (*z* > 3 or *z* < −3), the absolute number of SNPs that are significant in both analyses is indicated in the plot. As a null comparator, these overlap analyses were also performed with GWAS results from a study of educational attainment in 1.1 million individuals [26] downloaded from EMBL-EBI’s GWAS catalog [27]. The educational attainment study contains 10,098,325 SNPs, the SiGN study contains 10,156,805 SNPs. The overlap analysis was only done on the SNPs that are present in both datasets: the size of this overlapping set is 7,822,831 SNPs. For the overall comparisons per subtype, we considered all five GWAS. At each *z*-score threshold, we calculated the overall Jaccard index: the ratio (range between 0 and 1) of the number of SNPs significant in all five analyses to the number of SNPs significant in any analysis. See Fig. 3 for a graphical explanation of this method.

### Lookup of MEGASTROKE loci in the union and intersect GWAS

Recently, the MEGASTROKE consortium completed the largest GWAS in IS and its subtypes [2]. From this GWAS, we extracted the index SNPs of each genome-wide significant locus in each subtype. We then looked up these SNPs in our GWAS to compare effect sizes, resulting in 15 ORs per SNP (for each of the phenotype definitions in each of the subtypes). See Table S6 for the summary statistics of these lookups. If the reference allele in MEGASTROKE was not identical to the reference allele in SiGN, the inverse of the odds ratio (1/OR) was taken. We counted how often the intersect showed the most extreme OR, out of all 96 ORs (15 ORs per SNP, for the 32 SNPs that were reported in MEGASTROKE). To determine the probability of the number of times intersect was most extreme, under the null hypothesis that all phenotype definitions are just as likely to show the most extreme OR, we performed a binomial test in R [28].

### Replication of new genome-wide hits in MEGASTROKE

To assess all genome-wide significant loci instead of the individual SNPs, we performed clumping in PLINK 1.9 [22] (http://pngu.mgh.harvard.edu/purcell/plink/). We used all SNPs significant at *α* = 1 × 10^−5^ as index SNPs. We generated clumps for all other SNPs within 250 kb of the index SNP and in LD with the index SNP (*r*^2^ > 0.05). We kept clumps if the *p* value of the index SNP was lower than 5 × 10^−8^. From the genome-wide significant clumps, only the unique ones were kept (some clumps significantly associated to multiple case definitions). In the case of duplicates, the summary statistics for the analysis with the lowest *p* value were kept. Ambiguous SNPs were removed, and if the reference allele in MEGASTROKE was not identical to the reference allele in SiGN, we calculated the inverse of the odds ratio (1/OR). This resulted in a list of 14 unique SNPs. We checked for SNPs that are not in a locus that had already been reported as an associated locus in MEGASTROKE, resulting in a list of five new SNPs (two for SVS and three for CES), which we looked up for replication. To this end, we ran the MEGASTROKE GWAS again (European and trans-ancestry analysis per subtype using TOAST [29]) without the SiGN cohort, to ensure summary statistics independent from the discovery GWAS. We set Bonferroni corrected *p* value thresholds to adjust for the number of SNPs looked up for the phenotype in question, and for the number of GWAS it was looked up in (two, for the European and trans-ancestry analyses). We did a meta-analysis of the MEGASTROKE GWAS without SiGN, and the SiGN GWAS, for the three replicating SNPs only (Table S5). We performed meta-analysis in METAL [30], with the inverse-variance weighting scheme.

## Supplementary information

Supplemental Tables 1–5 & 8 + Figures 1–4 & 6

Table S6

Table S7

Figure S5
