# Supplementary material for: Alternate approach to stroke phenotyping identifies a genetic risk locus for small vessel stroke
Source: Eur J Hum Genet. 2020 Feb 11;28(7):963–72. doi: 10.1038/s41431-020-0580-5 (PMC7316747; doi:10.1038/s41431-020-0580-5)
Supplement: Supplementary file 1 — Supplemental Tables 1–5 & 8 + Figures 1–4 & 6 [file 41431_2020_580_MOESM1_ESM.docx]

**Supplemental table 1. Sample sizes**

| **LAS** |  |  |  |  |  |
| --- | --- | --- | --- | --- | --- |
| **Phenotype** | controls | cases | total | μ (case fraction) | control:case ratio |
| **TOAST** | 28,026 | 2,318 | 30,344 | 0.08 | 12.09 |
| **CCSc** | 28,026 | 1,565 | 29,591 | 0.05 | 17.91 |
| **CCSp** | 28,026 | 2,449 | 30,475 | 0.08 | 11.44 |
| **intersect** | 28,026 | 1,328 | 29,354 | 0.05 | 21.10 |
| **union** | 28,026 | 3,495 | 31,521 | 0.11 | 8.02 |
| **Symmetric difference** | 28,026 | 2,167 | 30,193 | 0.07 | 12.93 |
| **CES** |  |  |  |  |  |
| **Phenotype** | controls | cases | total | μ (case fraction) | control:case ratio |
| **TOAST** | 28,026 | 3,333 | 31,359 | 0.11 | 8.41 |
| **CCSc** | 28,026 | 3,000 | 31,026 | 0.10 | 9.34 |
| **CCSp** | 28,026 | 3,608 | 31,634 | 0.11 | 7.77 |
| **intersect** | 28,026 | 2,219 | 30,245 | 0.07 | 12.63 |
| **union** | 28,026 | 4,502 | 32,528 | 0.14 | 6.23 |
|  |  |  |  |  |  |
| **SVS** |  |  |  |  |  |
| **Phenotype** | controls | cases | total | μ (case fraction) | control:case ratio |
| **TOAST** | 28,026 | 2,631 | 30,657 | 0.09 | 10.65 |
| **CCSc** | 28,026 | 2,262 | 30,288 | 0.07 | 12.39 |
| **CCSp** | 28,026 | 2,419 | 30,445 | 0.08 | 11.59 |
| **intersect** | 28,026 | 1,548 | 29,574 | 0.05 | 18.10 |
| **union** | 28,026 | 3,480 | 31,506 | 0.11 | 8.05 |

**Supplemental table 2. Data overview**

| **Feature** | **number of individuals (percentage)** |
| --- | --- |
| **Age (SD)** | 63.93 (16.57) |
| **Chromosomal sex** | 21,152 XX (45%), 25,865 XY (55%) |
| **Hypertension** | 19,835 (54.8%) |
| **Diabetes Mellitus** | 6,846 (18.9% |
| **Atrial fibrillation** | 3,342 (17.5%) |
| **Coronary Artery Disease** | 6,228 (17.7%) |
| **Current smokers** | 6,391 (16.7%) |
| **Former smokers** | 13,203 (34.5%) |

**Supplemental table 3. Heritability estimates from BOLT-REML**

Heritabilities on the liability scale

| **Subtype** | **CCSc** | **CCSp** | **TOAST** | **intersect** | **union** | **symdif** |
| --- | --- | --- | --- | --- | --- | --- |
| **CES** | 0.194 | 0.174 | 0.183 | 0.275 | 0.139 | 0.146 |
| **LAS** | 0.258 | 0.164 | 0.164 | 0.263 | 0.126 | 0.144 |
| **SVS** | 0.315 | 0.173 | 0.154 | 0.316 | 0.116 | 0.142 |

Standard errors of the heritabilities on the liability scale

| **Subtype** | **CCSc** | **CCSp** | **TOAST** | **intersect** | **union** | **symdif** |
| --- | --- | --- | --- | --- | --- | --- |
| **CES** | 0.013 | 0.011 | 0.012 | 0.017 | 0.009 | 0.016 |
| **LAS** | 0.023 | 0.016 | 0.017 | 0.029 | 0.012 | 0.017 |
| **SVS** | 0.029 | 0.016 | 0.015 | 0.024 | 0.012 | 0.020 |

**Supplemental table 4. P-values of heritability differences**

Significant differences at α = 0.005 as determined by t-test (corresponding to a Bonferroni correction for 10 comparisons per subtype), are indicated in the tables below in bold. Symdif = symmetric difference.

| **CES** | CCSc | CCSp | TOAST | intersect | union | symdif |
| --- | --- | --- | --- | --- | --- | --- |
| CCSc | 1.0 | 2.6e-01 | 5.3e-01 | **2.2e-04** | **6.5e-04** | 2.2e-02 |
| CCSp |  | 1.0 | 6.2e-01 | **1.4e-06** | 1.5e-02 | 1.5e-01 |
| TOAST |  |  | 1.0 | **1.3e-05** | **3.6e-03** | 6.8e-02 |
| intersect |  |  |  | 1.0 | **5.4e-12** | **7.0e-08** |
| union |  |  |  |  | 1.0 | 7.2e-01 |
| symdif |  |  |  |  |  | 1.0 |

| **LAS** | CCSc | CCSp | TOAST | intersect | union | symdif |
| --- | --- | --- | --- | --- | --- | --- |
| CCSc | 1.0 | **8.5e-04** | **1.0e-03** | 8.8e-01 | **3.9e-07** | **8.2e-05** |
| CCSp |  | 1.0 | 9.9e-01 | **2.4e-03** | 5.7e-02 | 3.9e-01 |
| TOAST |  |  | 1.0 | **2.7e-03** | 7.0e-02 | 4.1e-01 |
| intersect |  |  |  | 1.0 | **9.0e-06** | **3.5e-04** |
| union |  |  |  |  | 1.0 | 4.1e-01 |
| symdif |  |  |  |  |  | 1.0 |

| **SVS** | CCSc | CCSp | TOAST | intersect | union | symdif |
| --- | --- | --- | --- | --- | --- | --- |
| CCSc | 1.0 | **2.0e-05** | **9.3e-07** | 9.8e-01 | **2.7e-10** | **8.7e-07** |
| CCSp |  | 1.0 | 3.7e-01 | **6.1e-07** | 3.7e-03 | 2.1e-01 |
| TOAST |  |  | 1.0 | **8.5e-09** | 4.7e-02 | 6.2e-01 |
| intersect |  |  |  | 1.0 | **5.4e-14** | **1.7e-08** |
| union |  |  |  |  | 1.0 | 2.6e-01 |
| symdif |  |  |  |  |  | 1.0 |

**Supplemental table 5. Meta-analysis of replicated SNPs with MEGASTROKE**

Results from METAL. CHR = chromosome, POS = position, SNP = rs-id of SNP, A1 = allele 1, A2 = allele 2, Frq = frequency of allele 2, FrqSE = standard error of Frq, MinFrq = minimum Frq, MaxFrq = maximum Frq, Beta = coefficient, SE = standard error of Beta, P = P-value, Dir = direction of effect in all analyses

| CHR | POS | SNP | A1 | A2 | Frq | FrqSE | MinFrq | MaxFrq | Beta | SE | P | Dir |
| --- | --- | --- | --- | --- | --- | --- | --- | --- | --- | --- | --- | --- |
| 4 | 114401929 | rs10029218 | a | g | 0.122 | 0.0003 | 0.1223 | 0.126 | 0.0168 | 0.0029 | 4.30E-09 | ++ |
| 12 | 112059557 | rs11065979 | t | c | 0.418 | 0.001 | 0.4176 | 0.4302 | 0.013 | 0.0022 | 3.04E-09 | ++ |
| 16 | 56340223 | rs3790099 | c | g | 0.845 | 0.0002 | 0.8432 | 0.8454 | -0.0206 | 0.0035 | 5.64E-09 | -- |

**Supplemental table 6. Summary statistics for previously known associations in the analyses performed in this study**

**See attached files Table-S6.xlsx**

**Supplemental Table 7. Summary statistics for previously known associations in the MEGASTROKE study**

**See attached files Table-S7.xlsx**

**Supplemental Table 8. Genomic regions removed before heritability estimation**

| Chromosome | Start (Mb) | End (Mb) | Name |
| --- | --- | --- | --- |
| 6 | 25.8 | 36 | MHC |
| 8 | 6 | 16 | inversion |
| 17 | 40 | 45 | inversion |

**Supplemental fig 1. QQ plots and Manhattan plots**

Quality control plots for all GWAS (symdif = symmetric difference) (A) QQ-plot stratified by imputation quality (assessed by INFO score). Inflation factor lambda is indicated in the plot. (B) QQ-plot stratified by minor allele frequency (MAF). Inflation factor lambda is indicated in the plot. (C) Manhattan plot


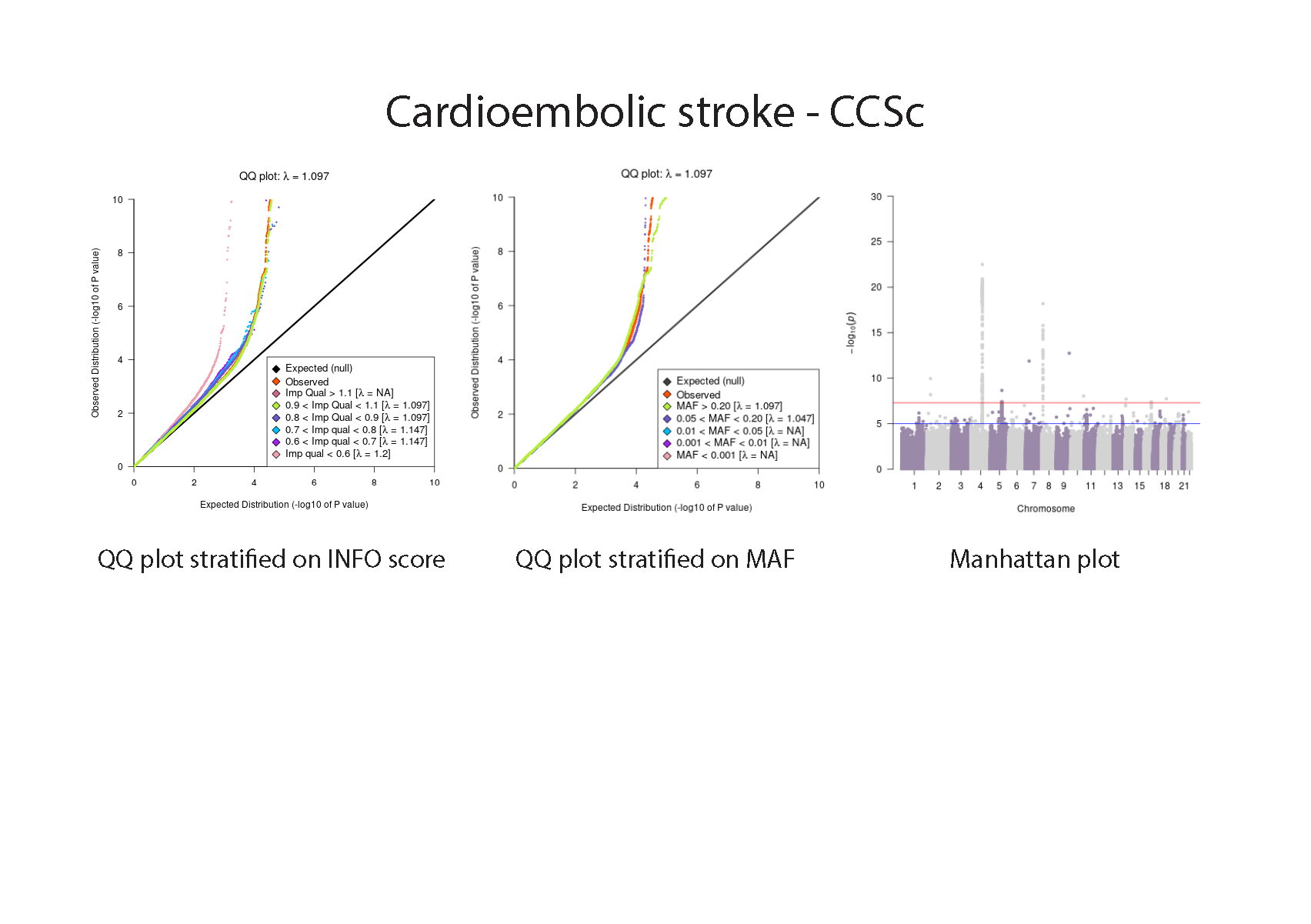


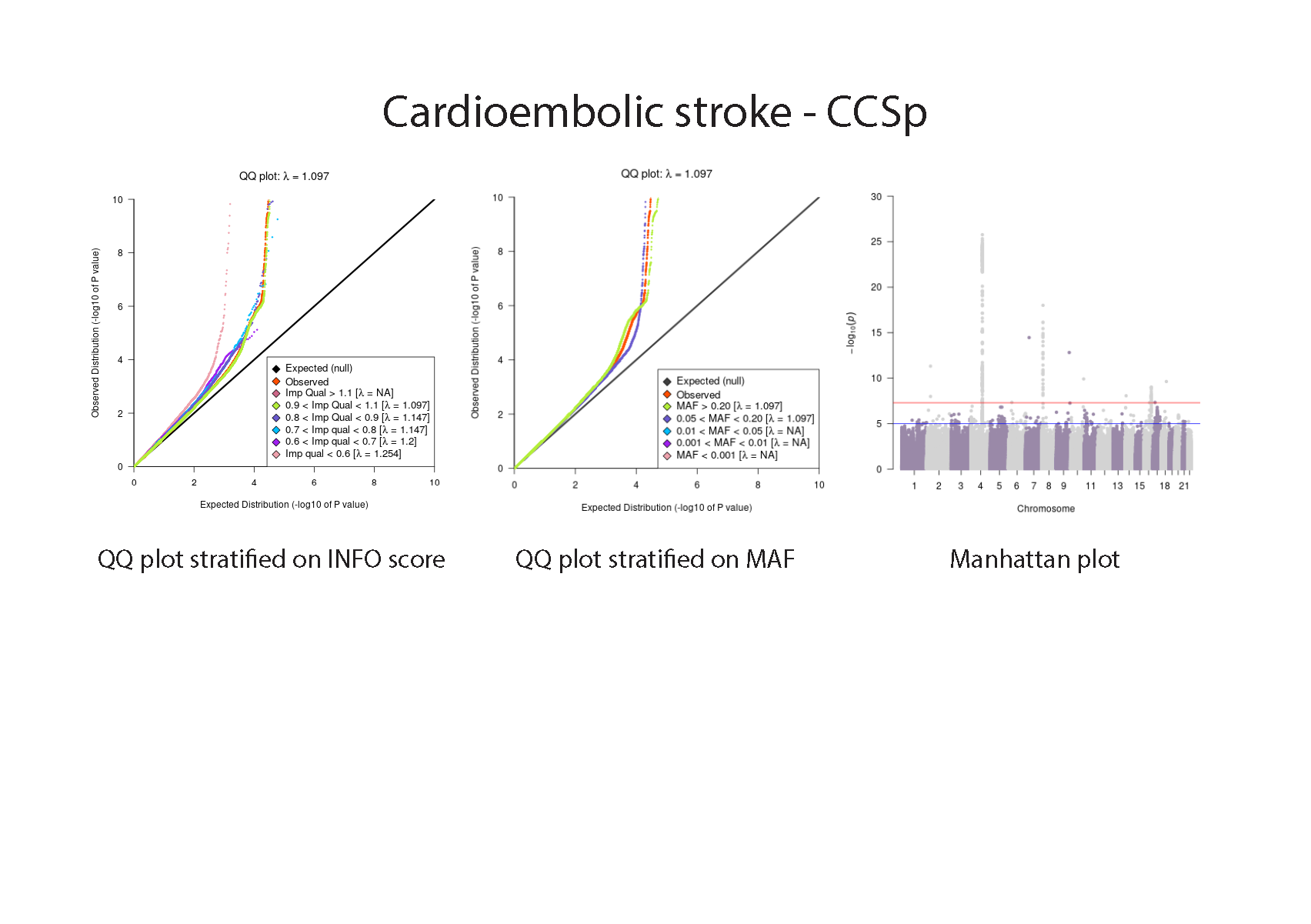


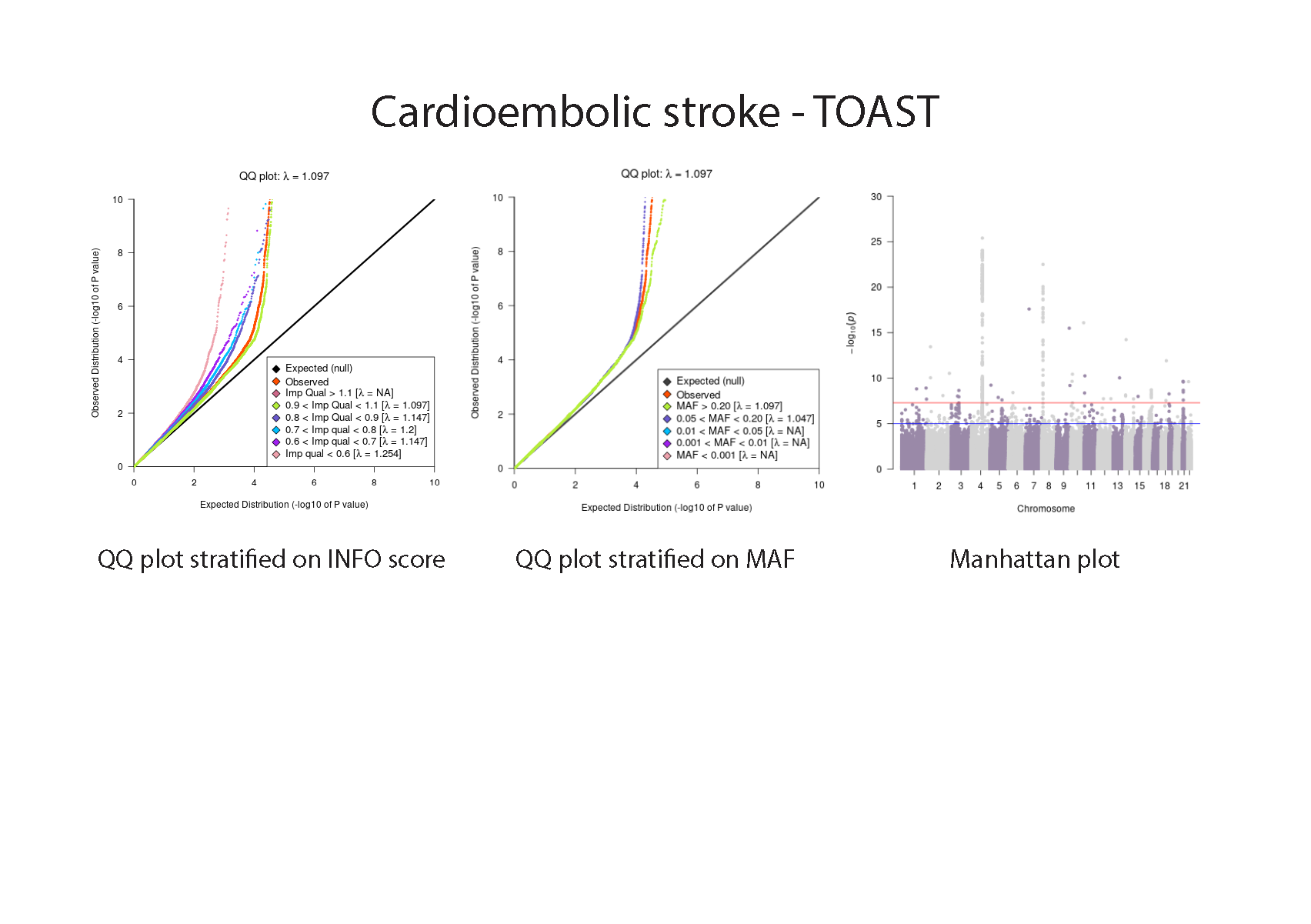


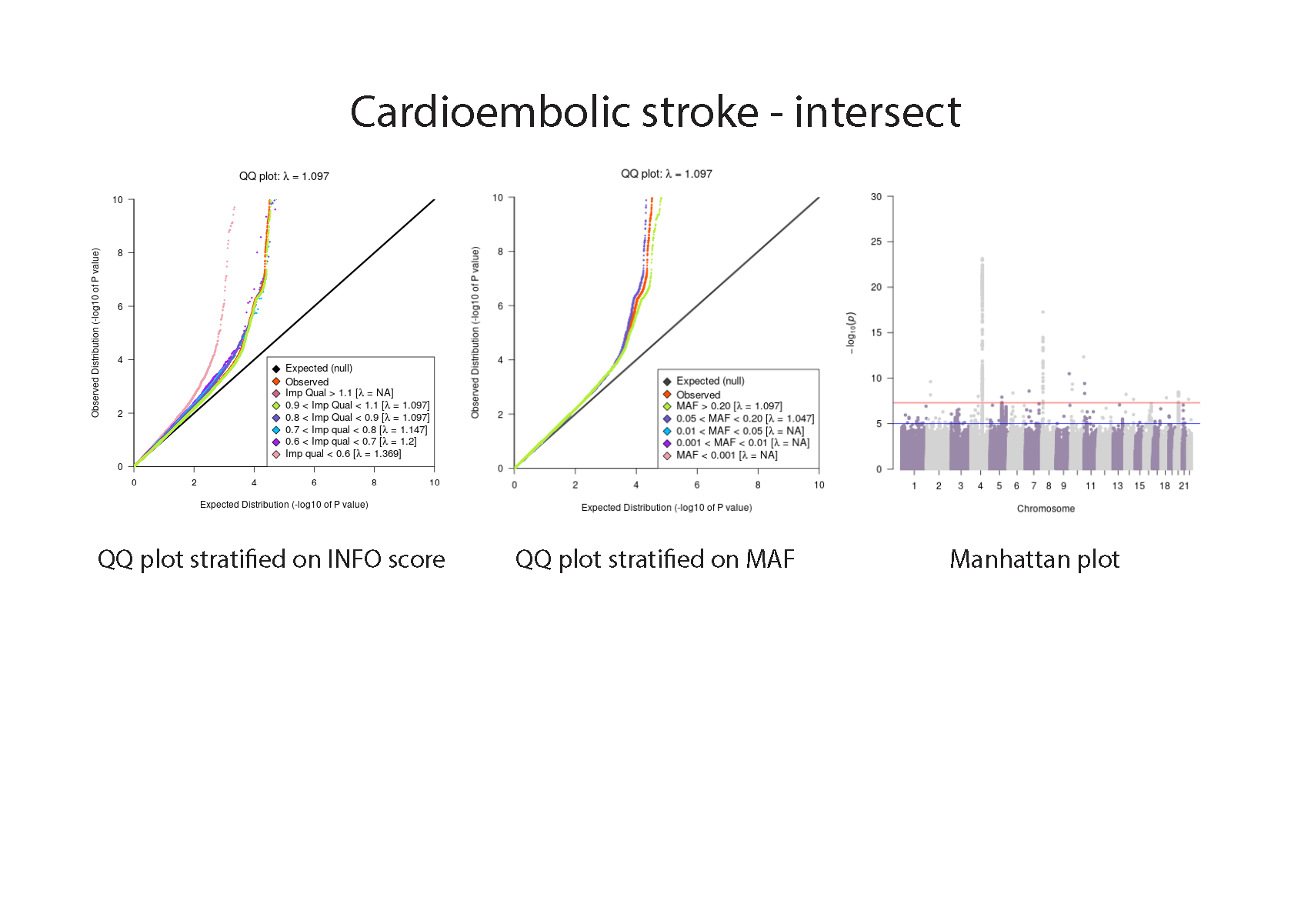


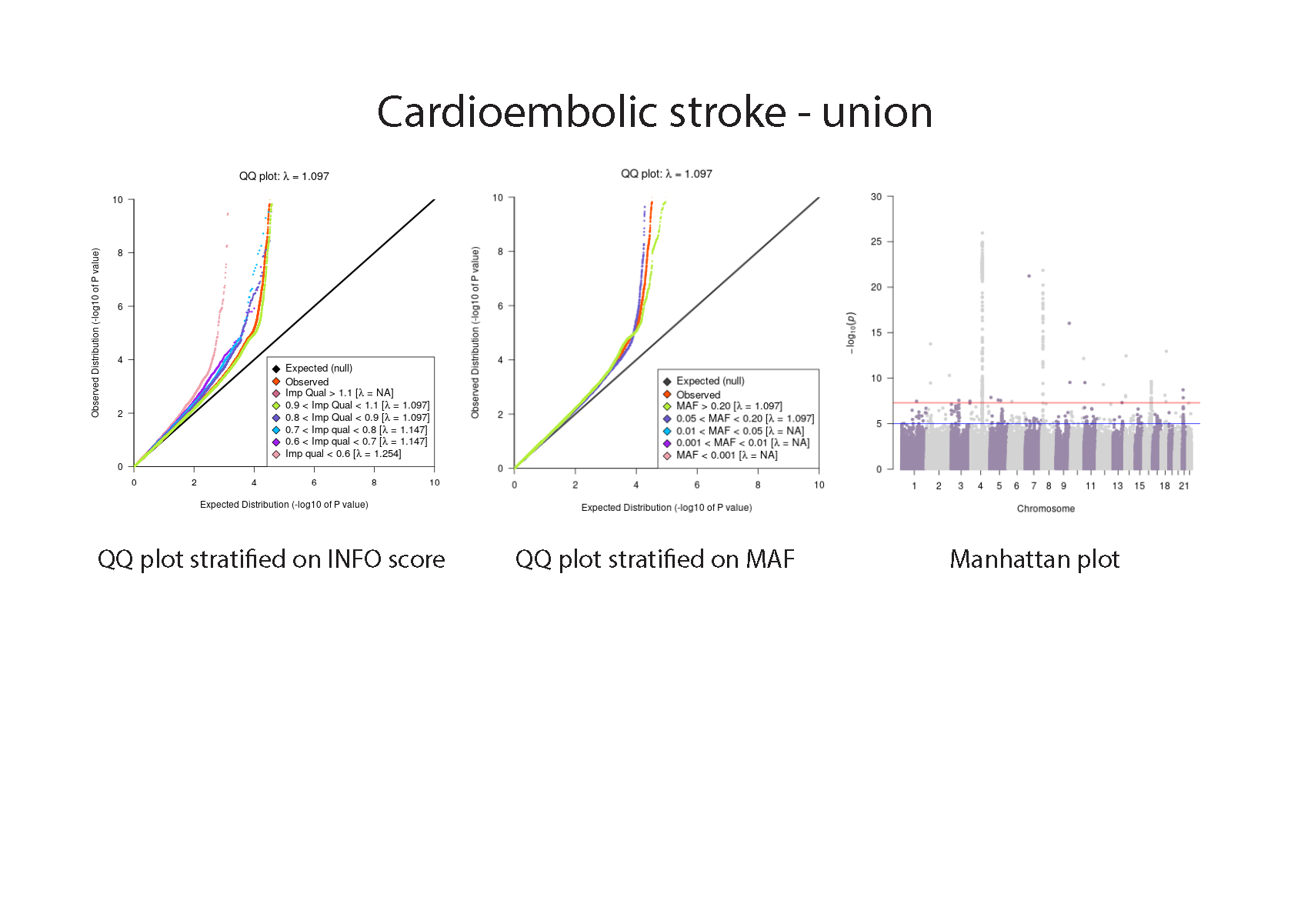


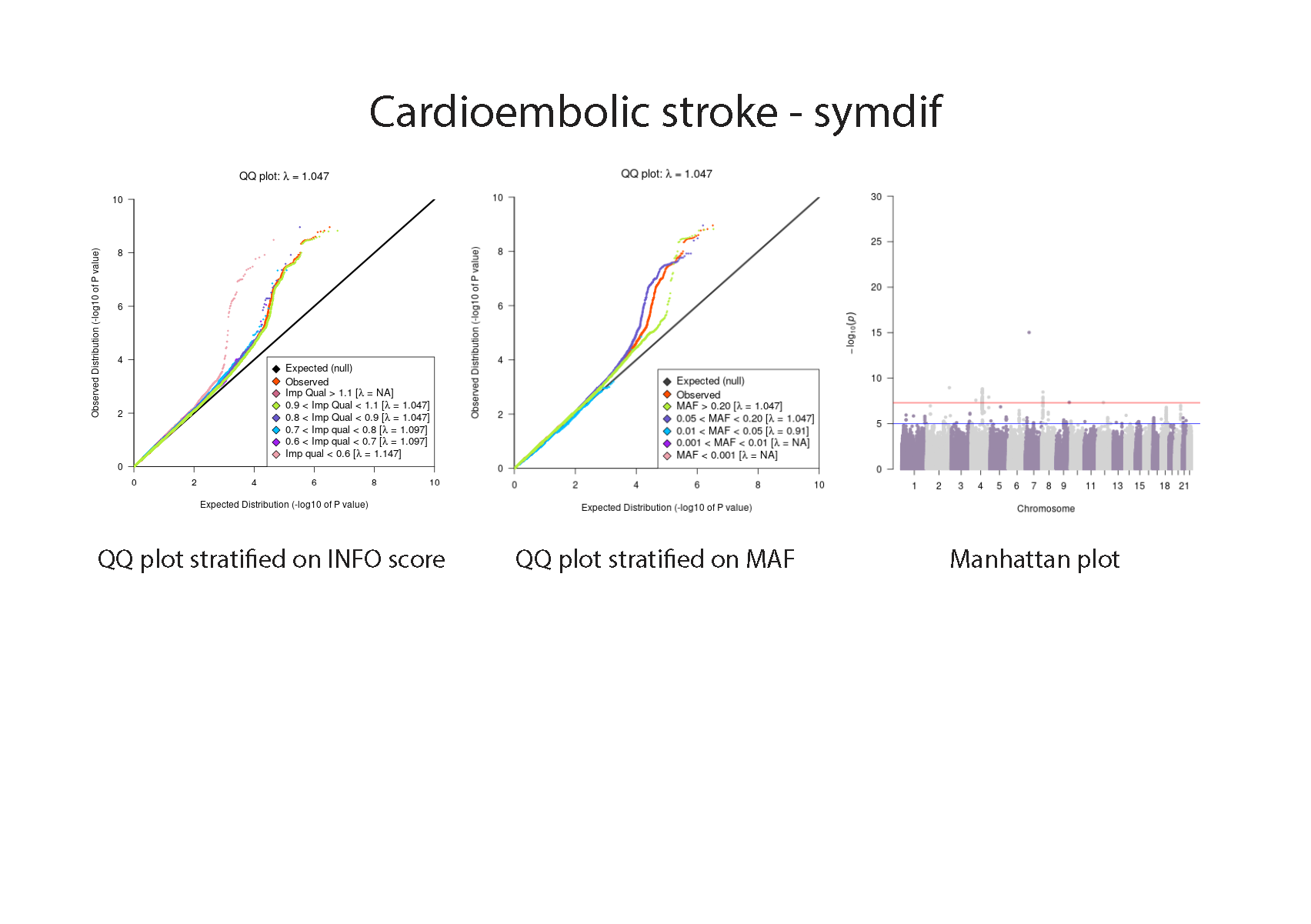


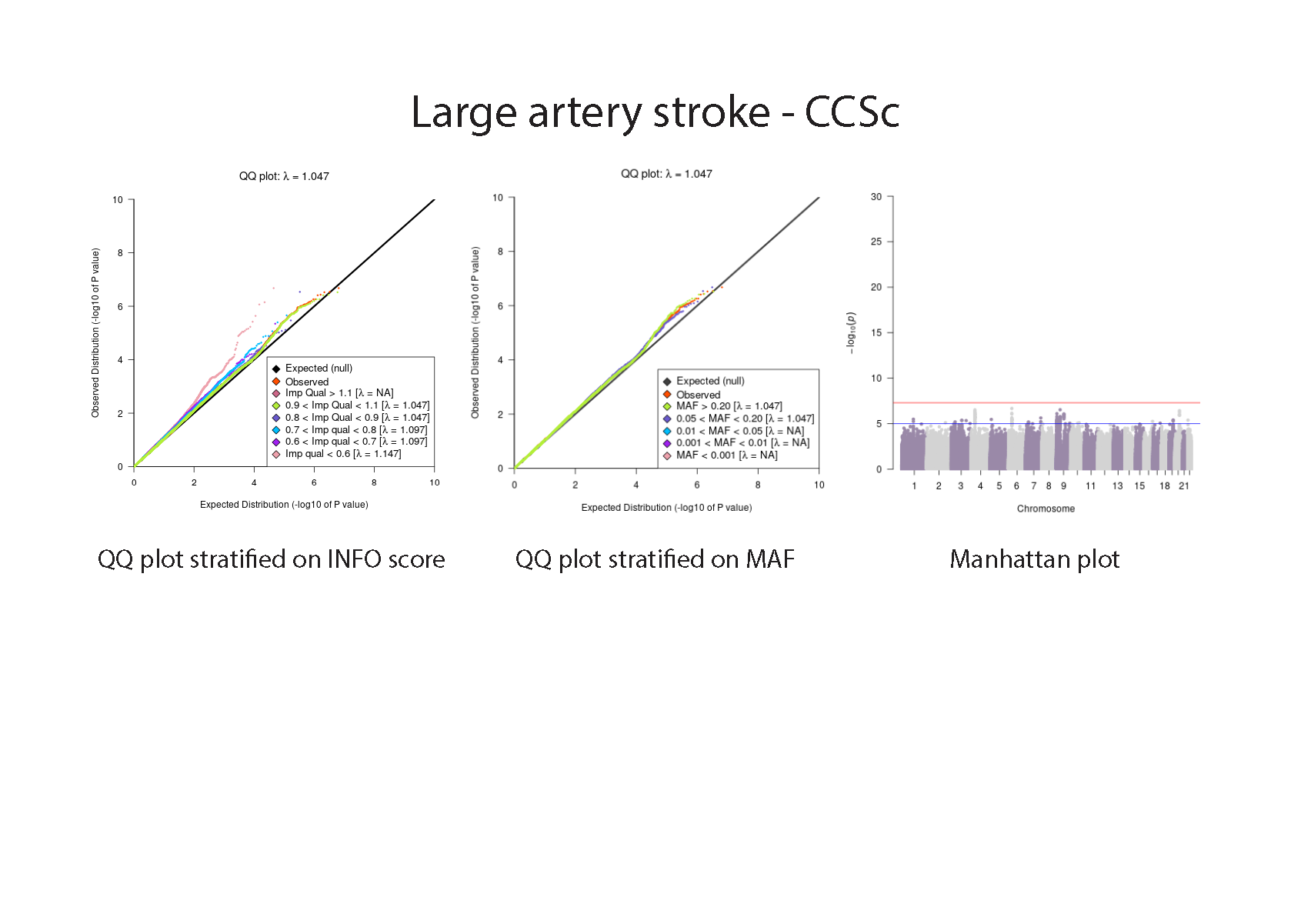


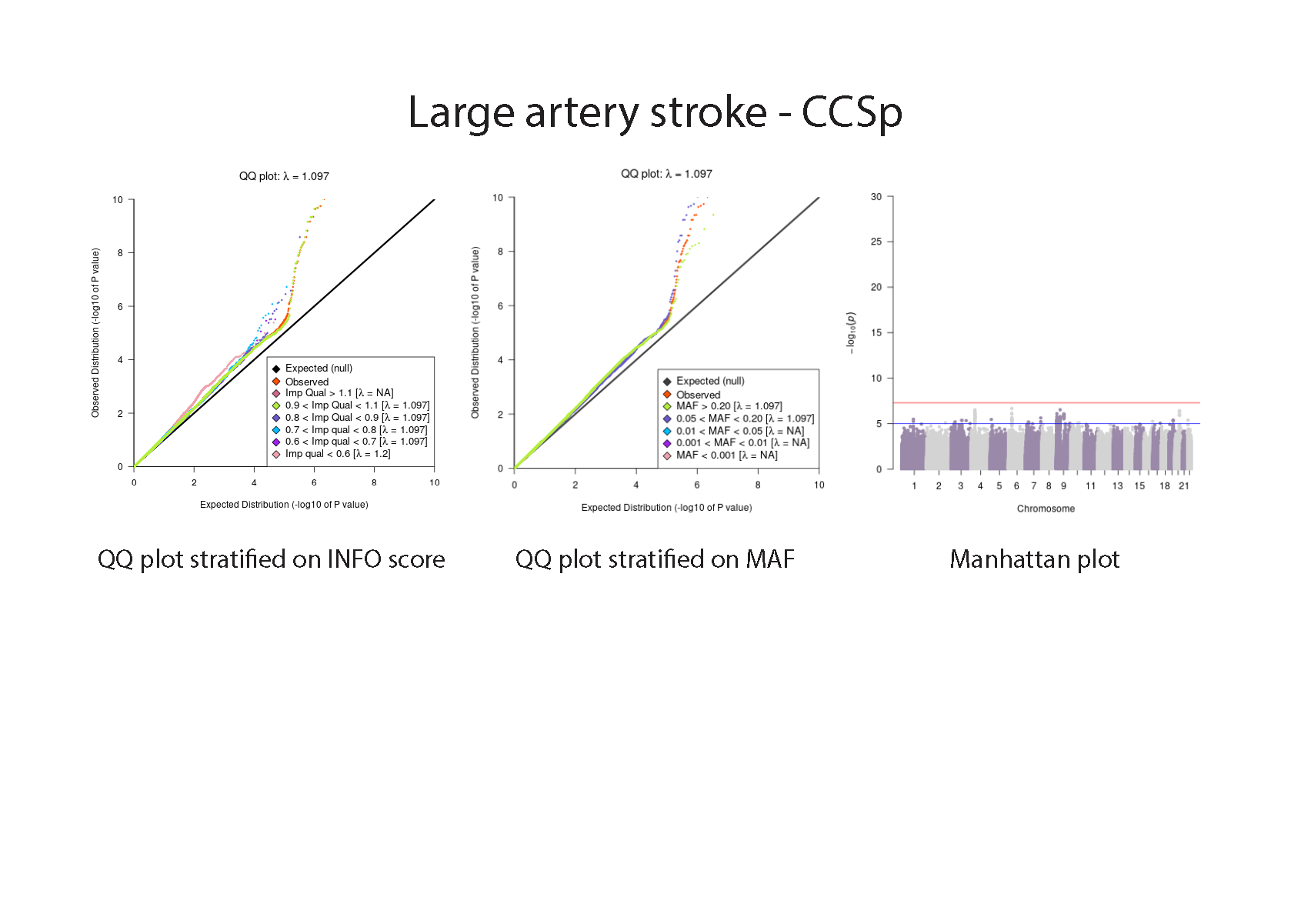


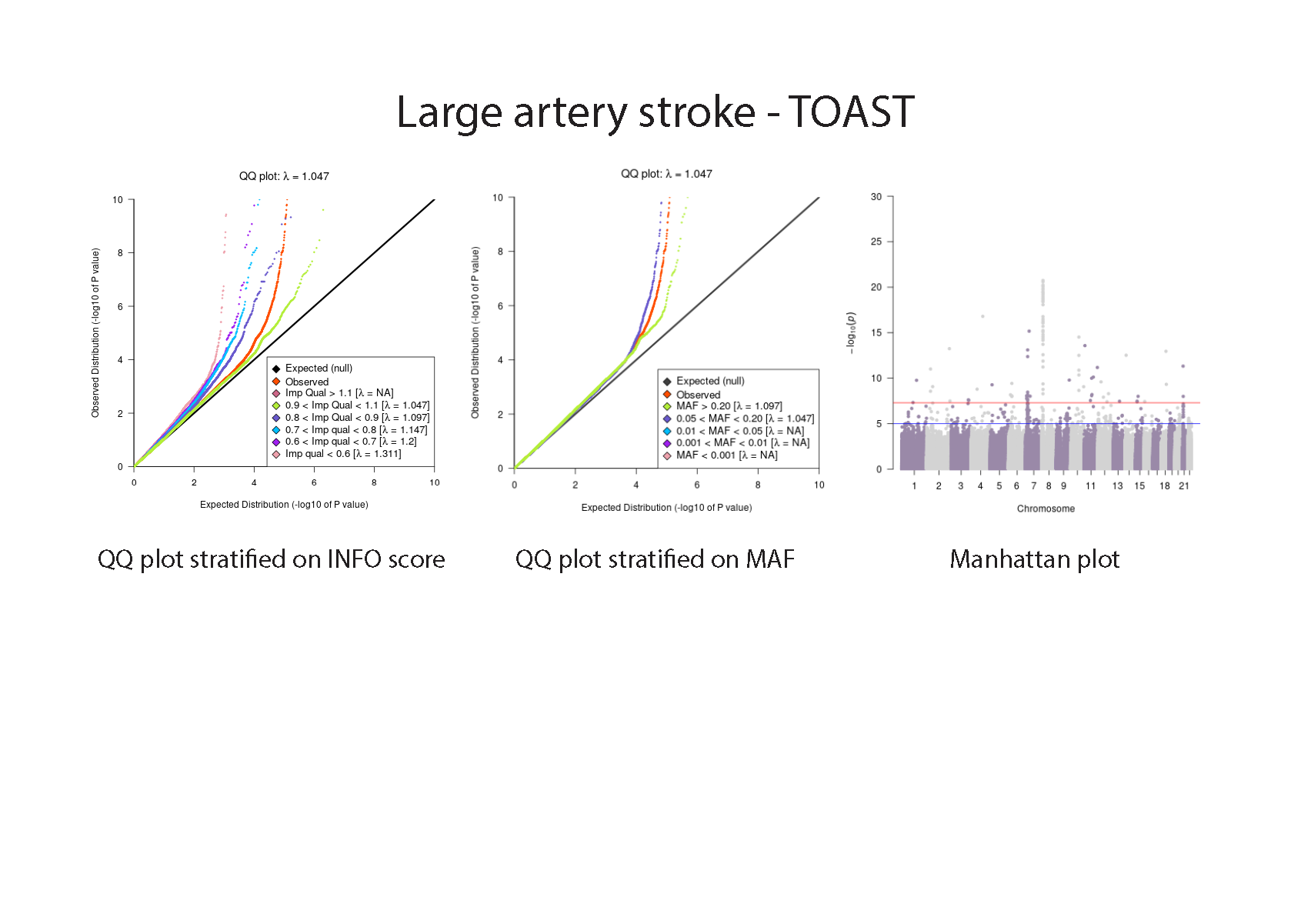


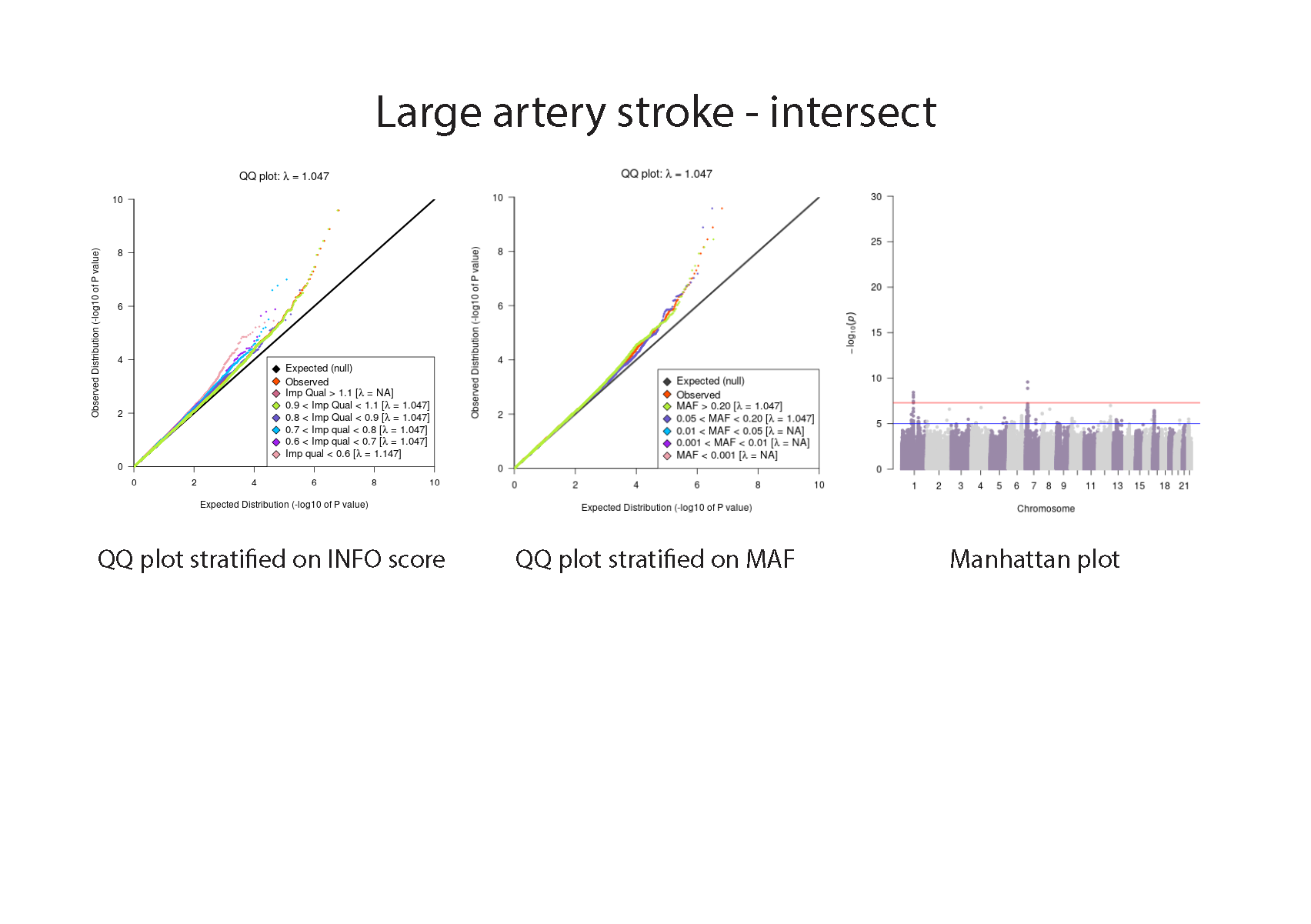


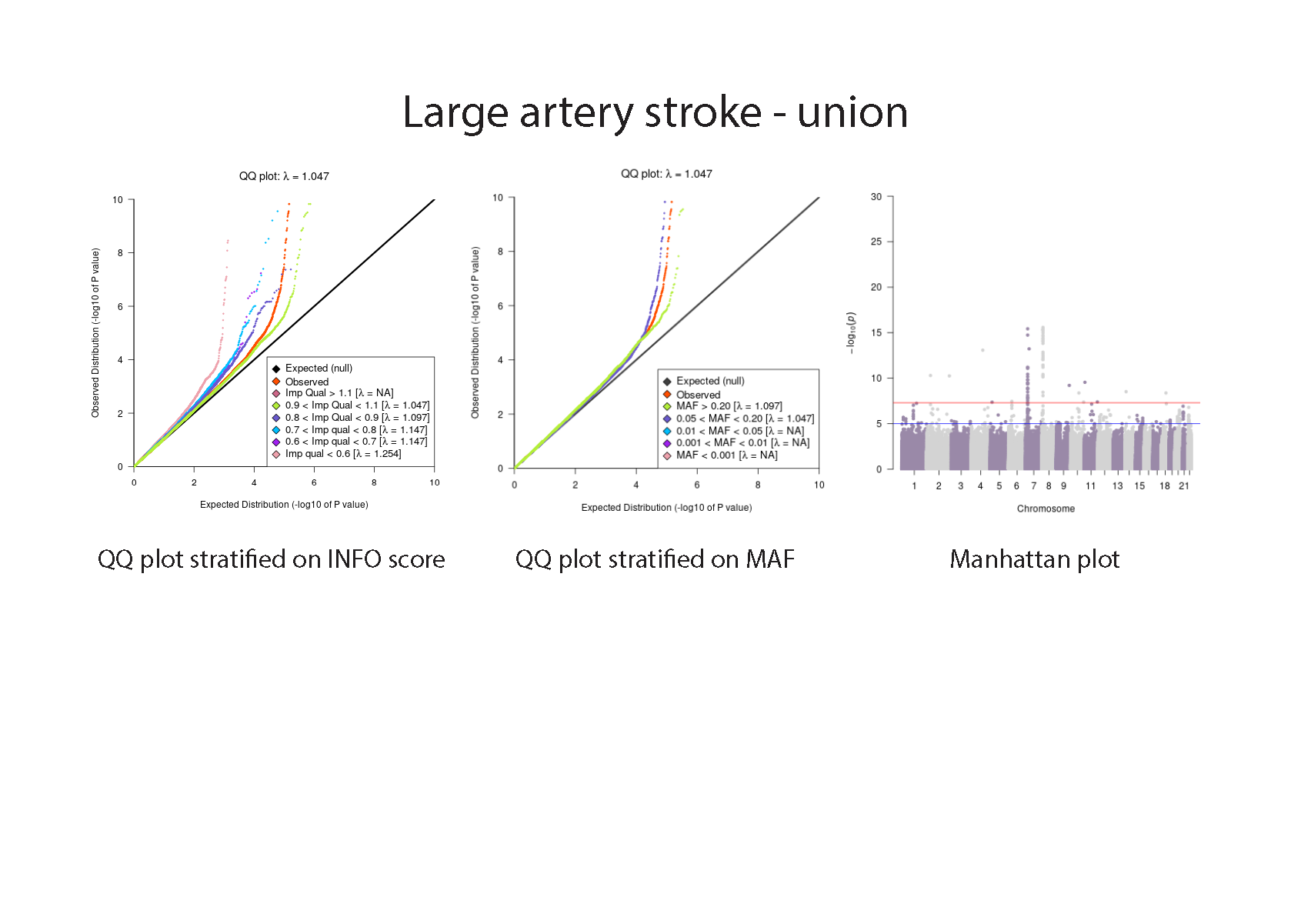


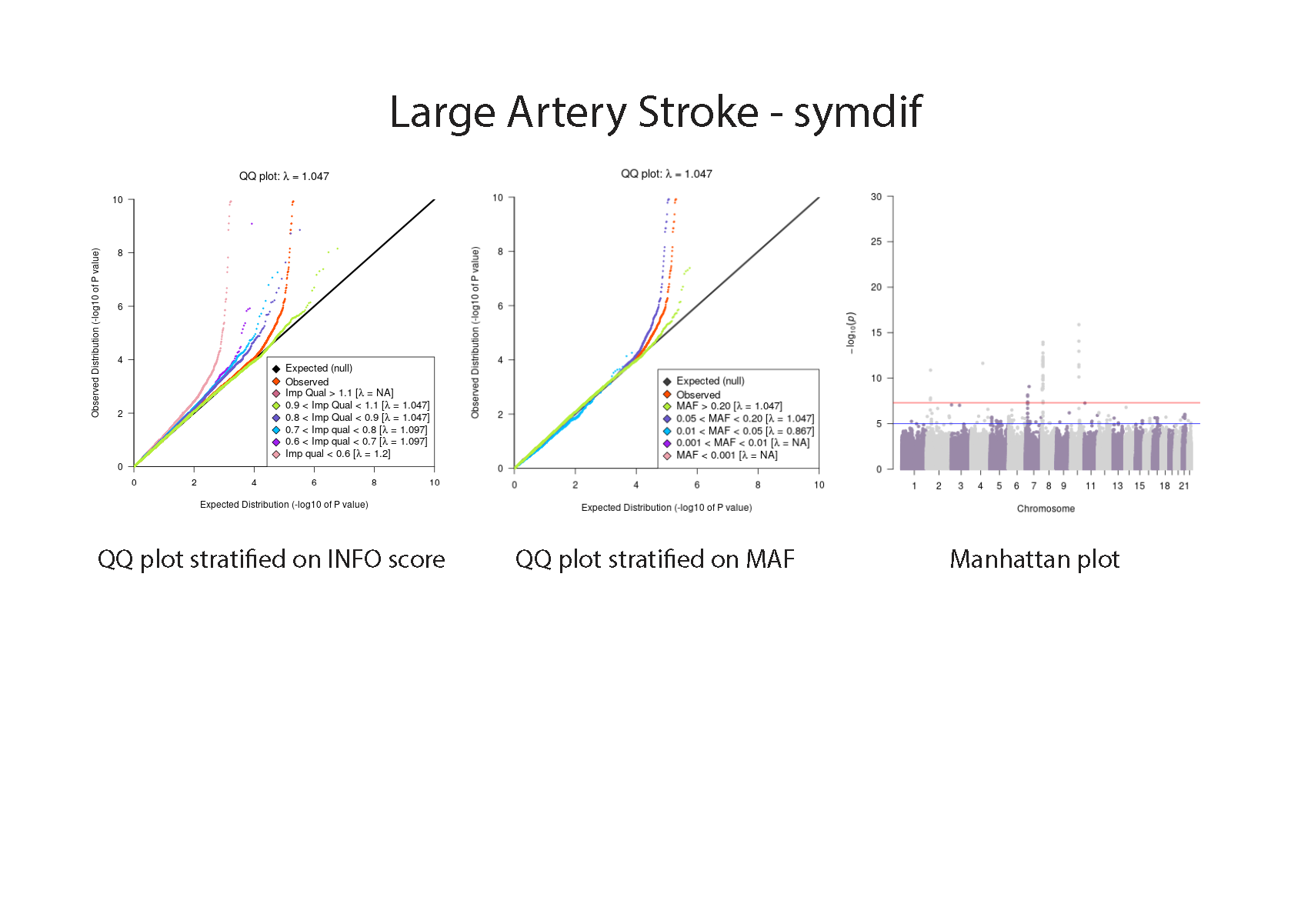


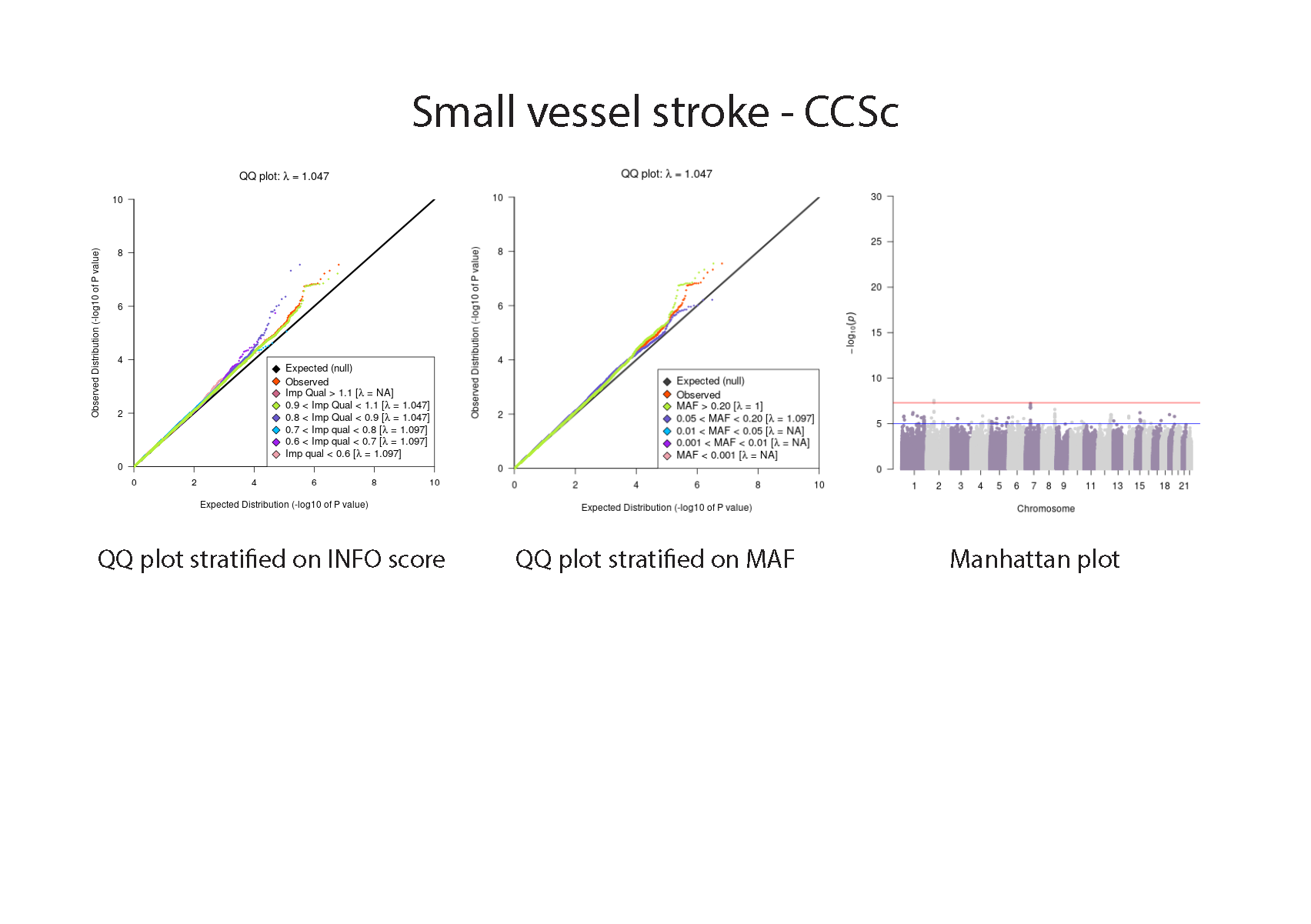


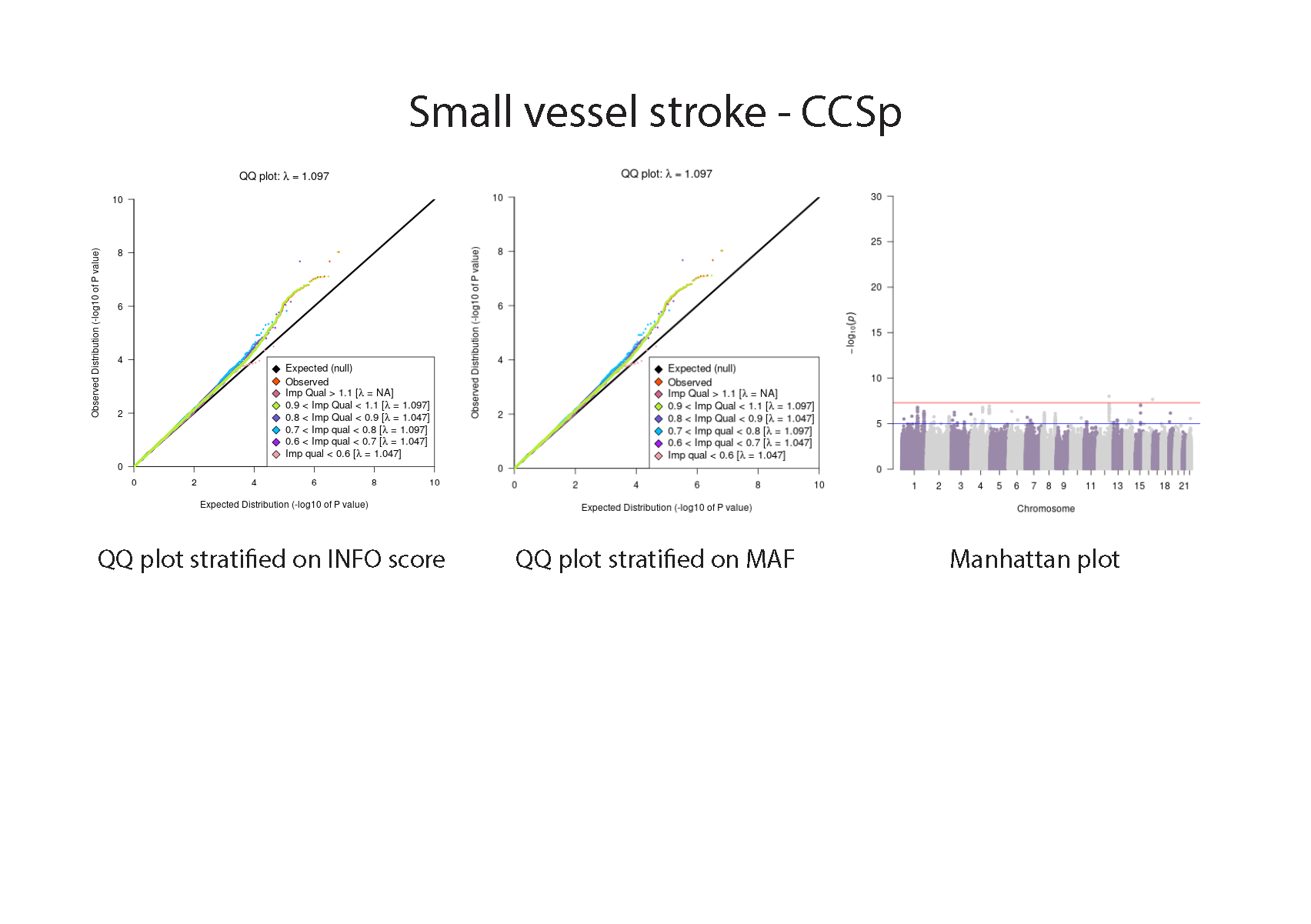


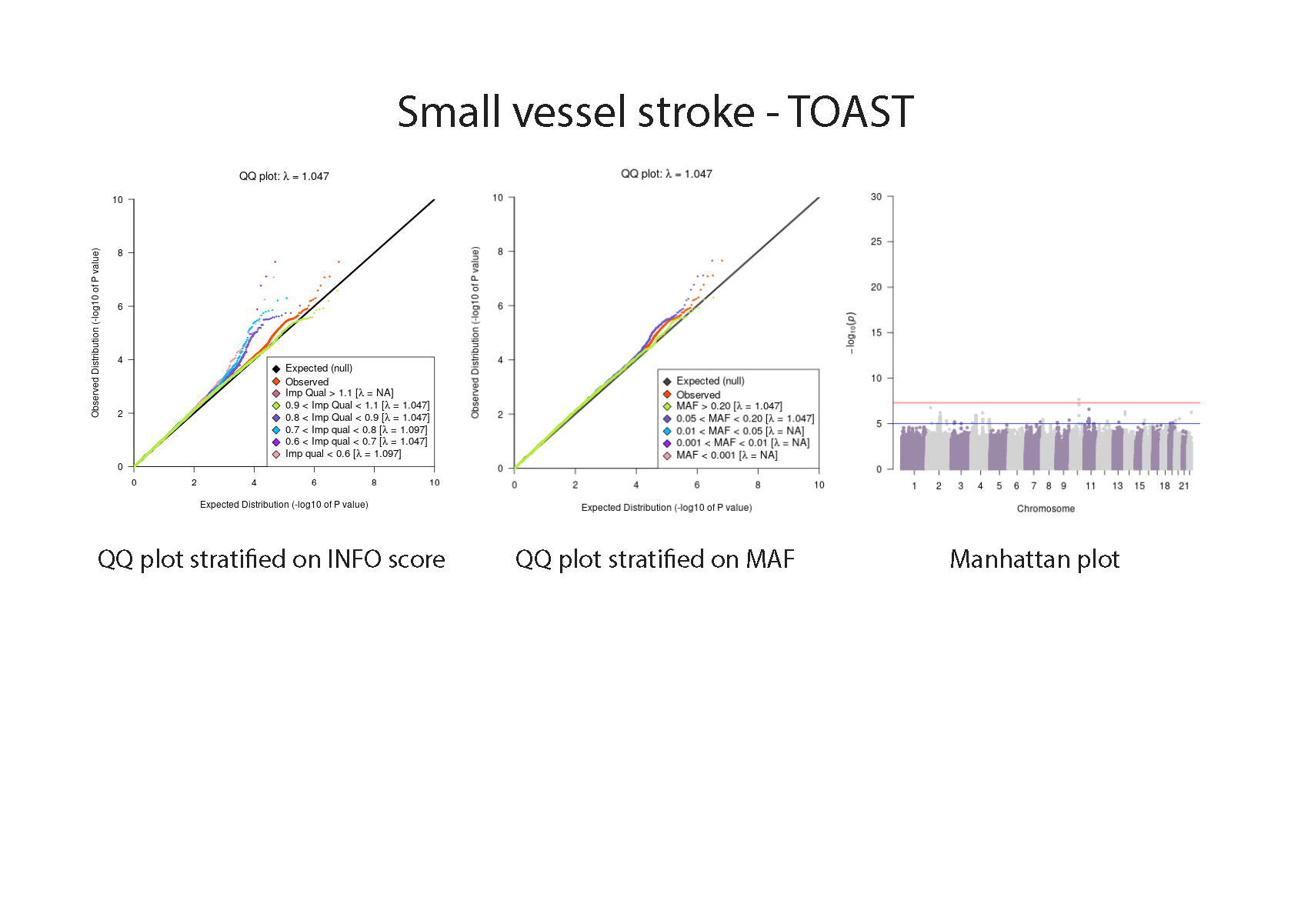


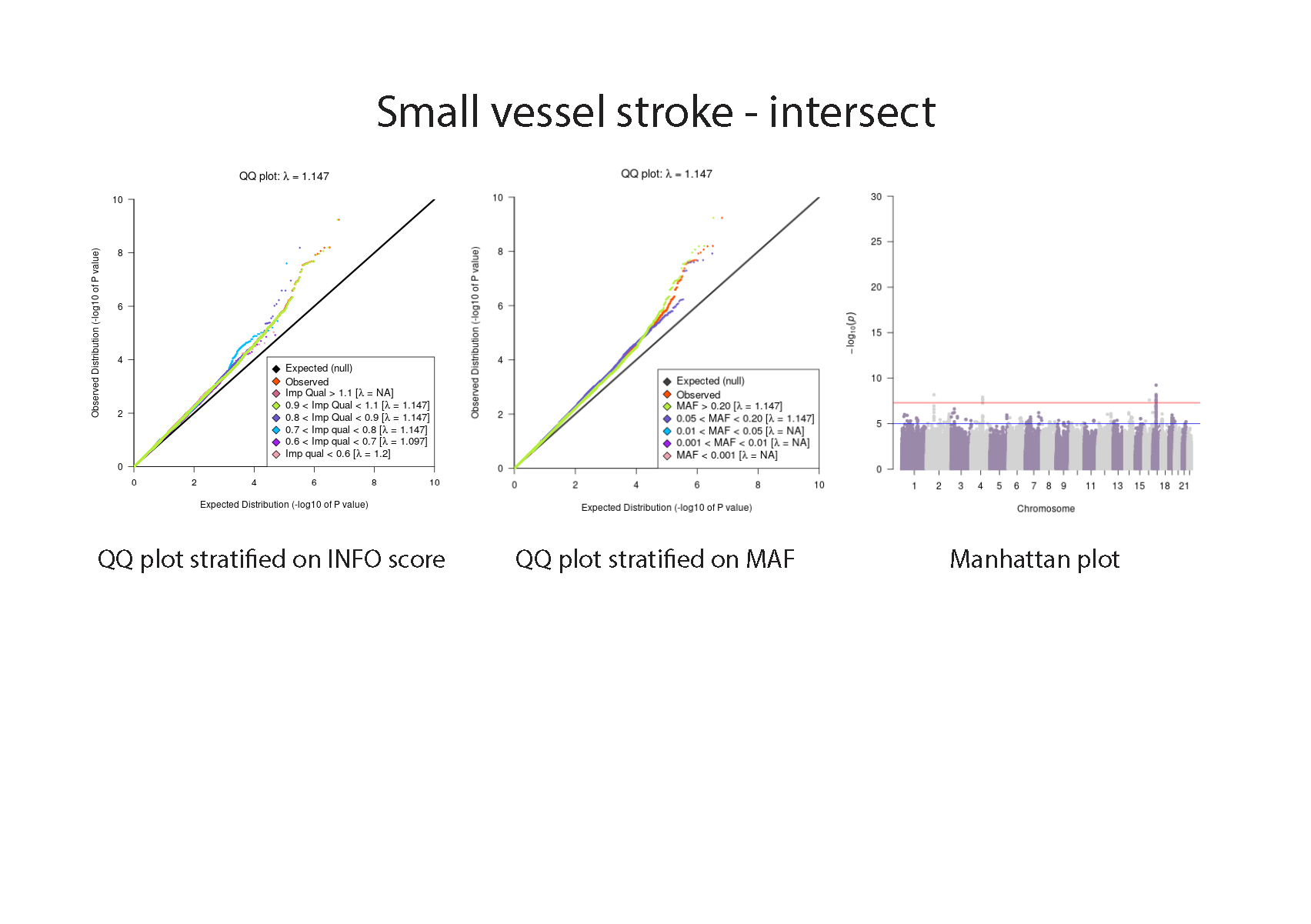


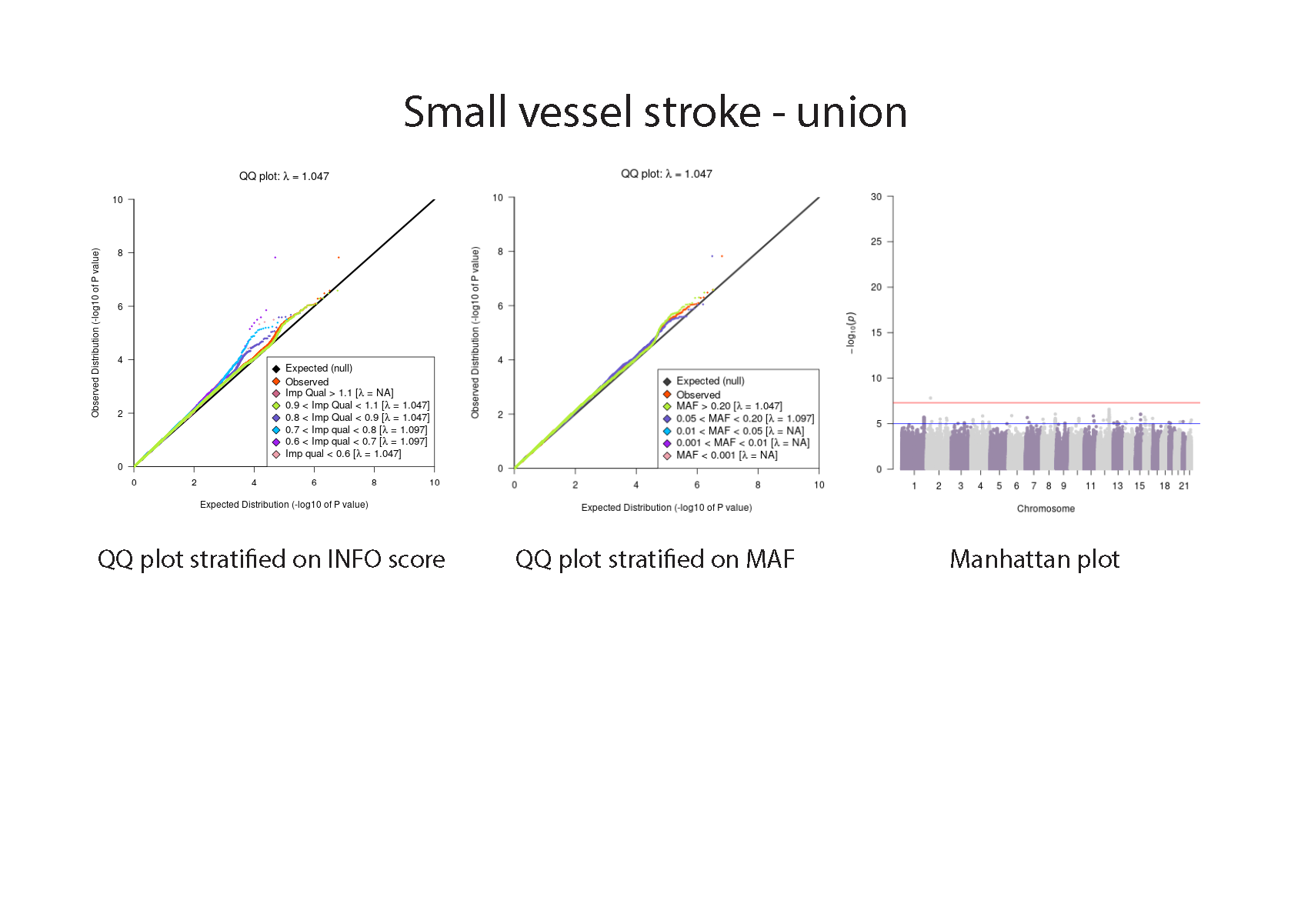


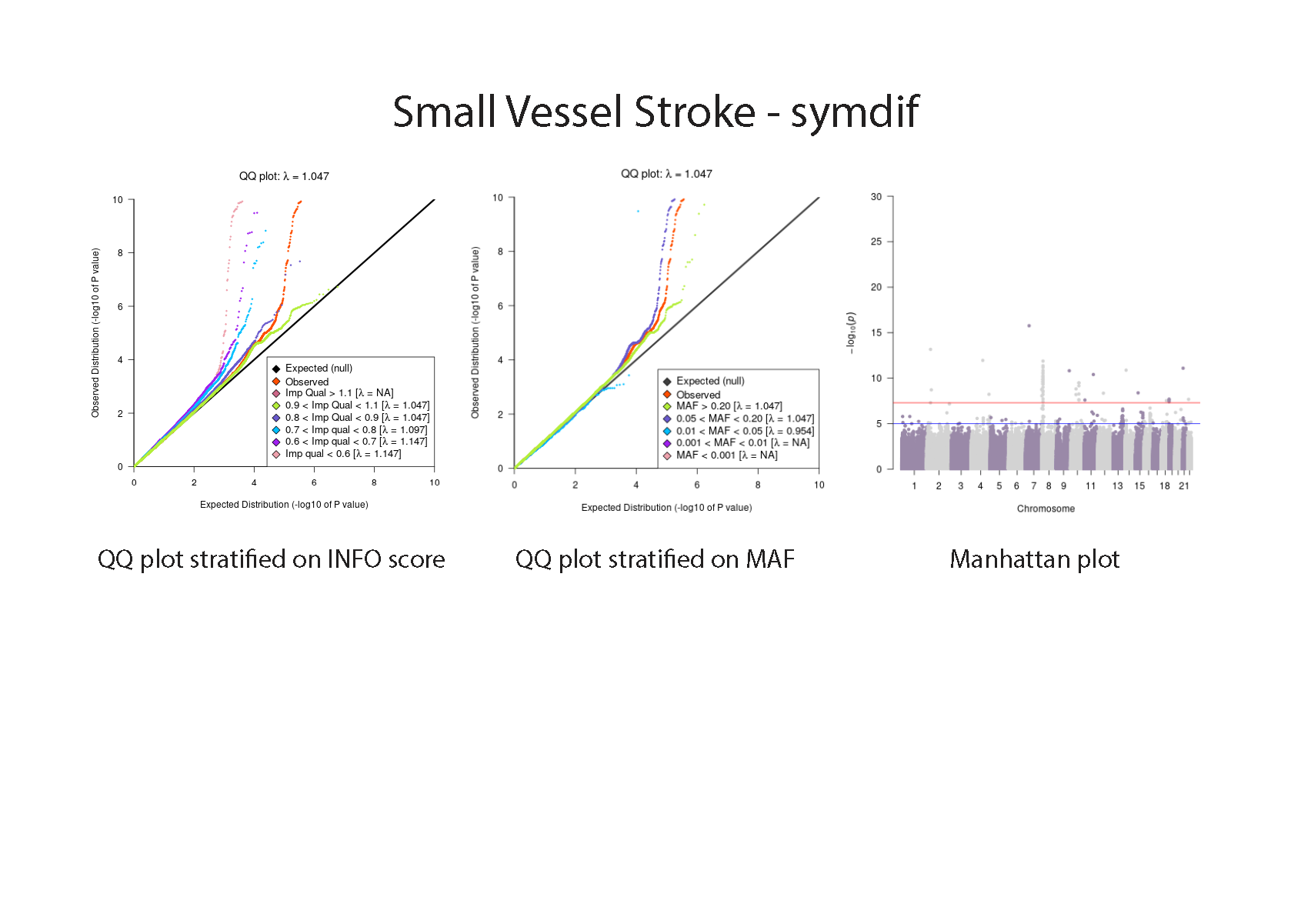


S**upplemental figure 2. Overlap analysis with cognitive performance as reference**


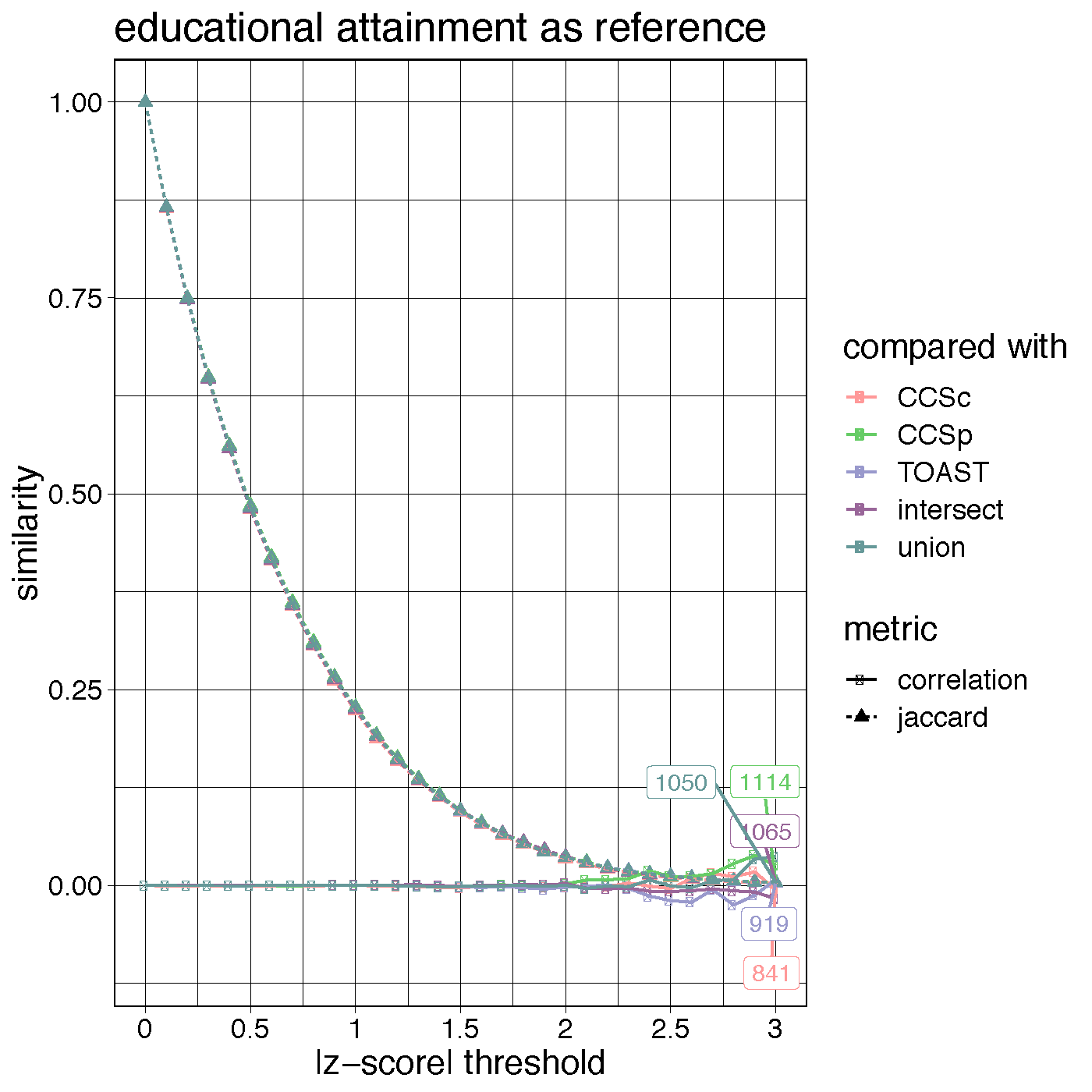

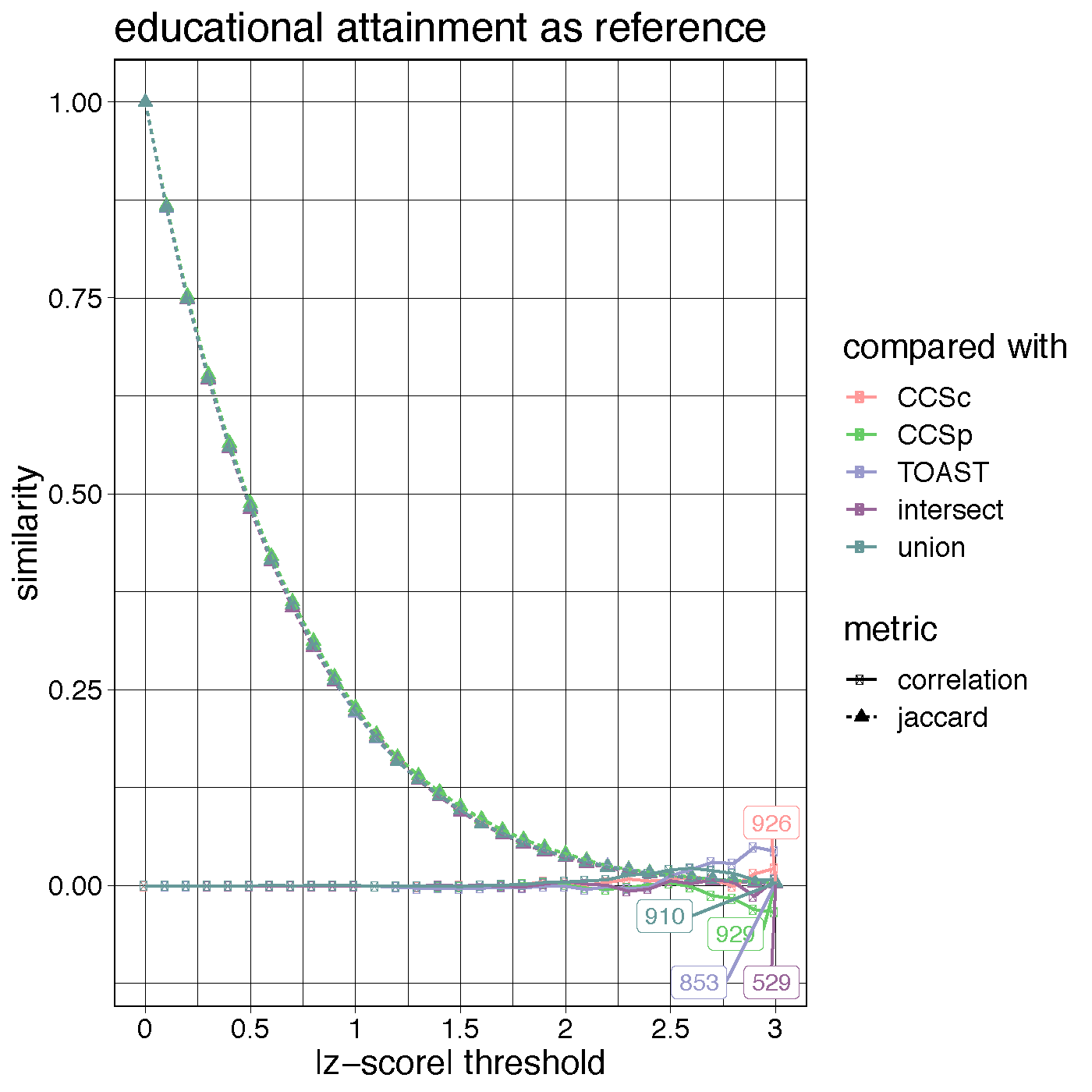

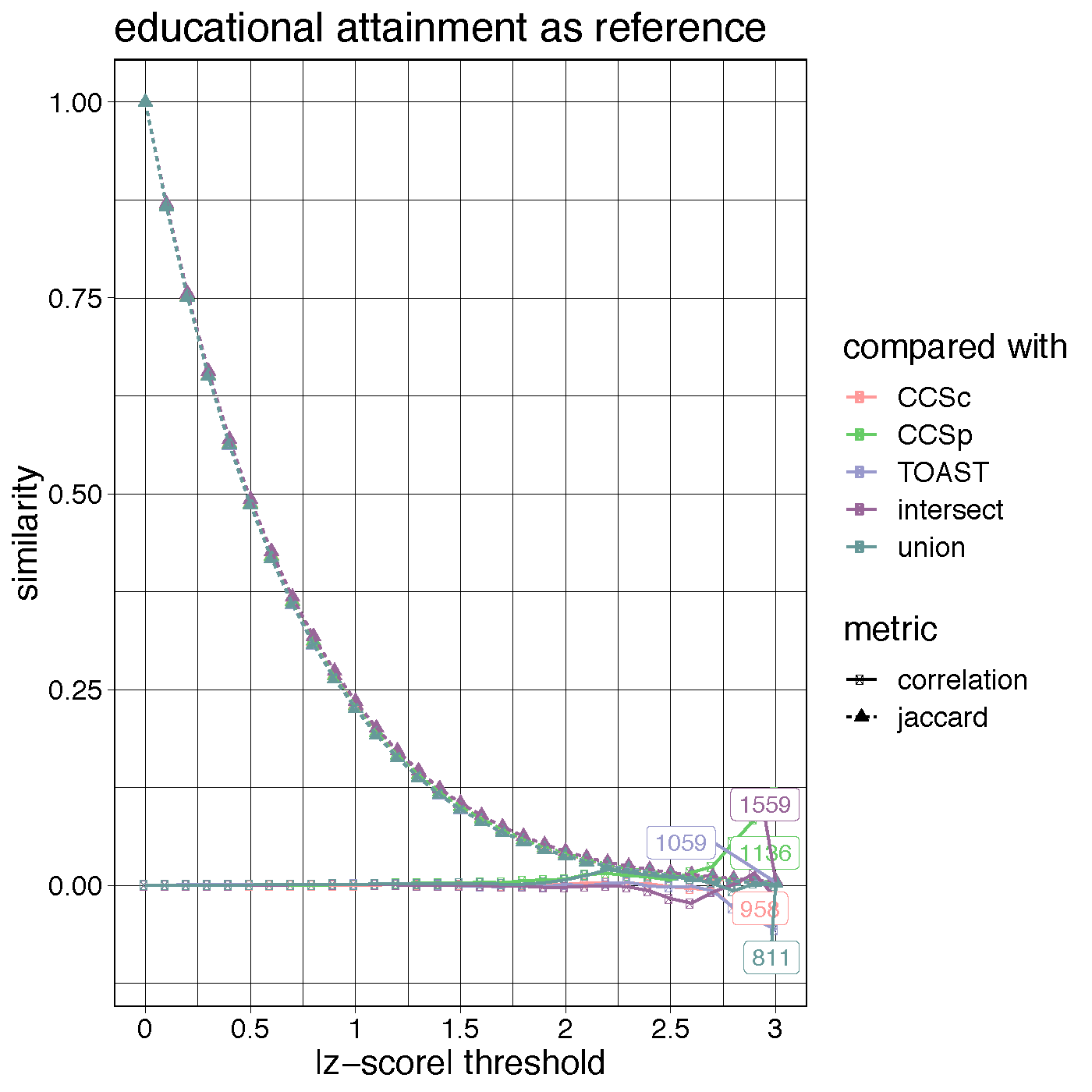


Cardioembolic stroke Large artery stroke Small vessel stroke

**Supplemental figure 3. Overlap plot LAS & SVS**

LAS


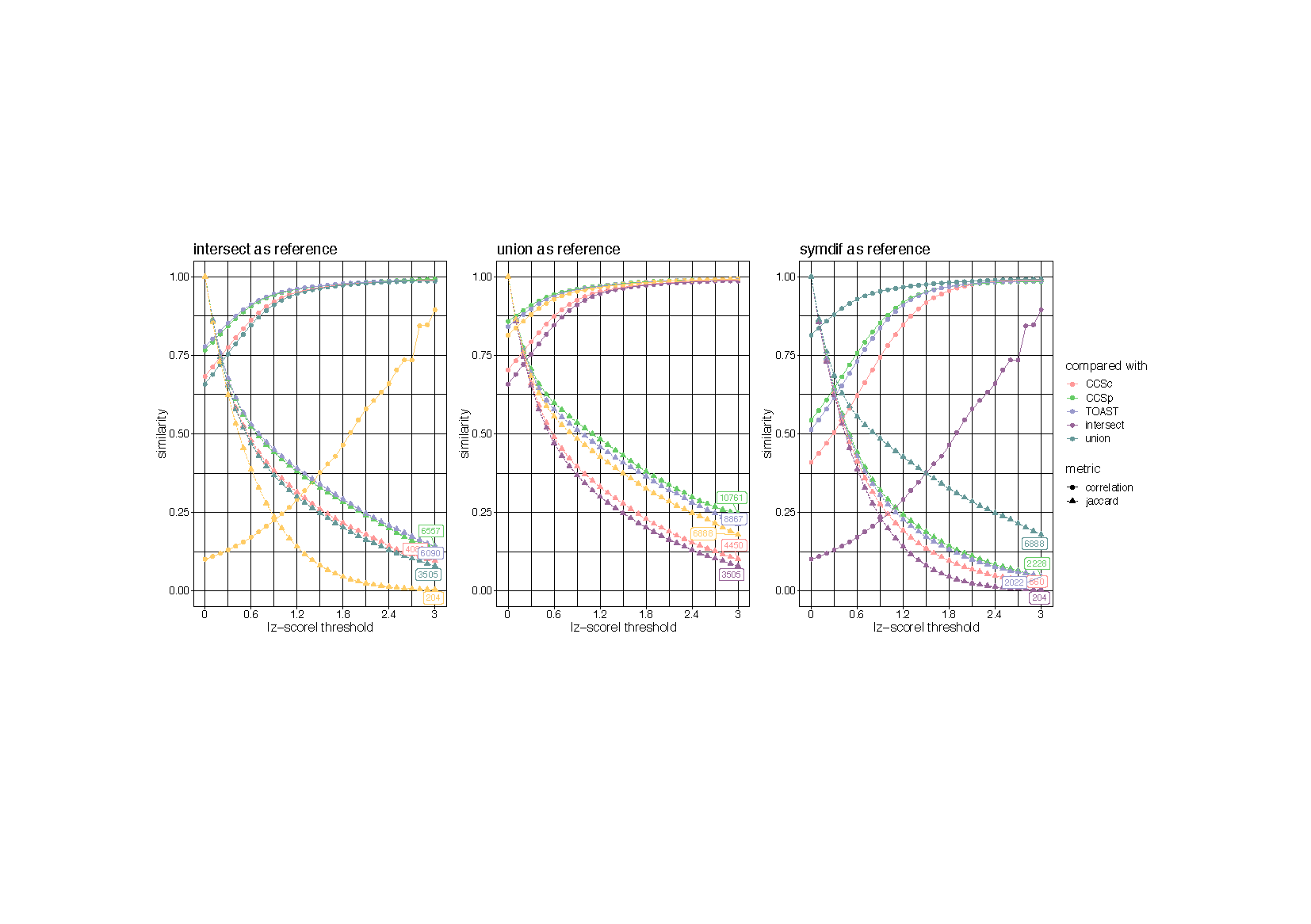


SVS


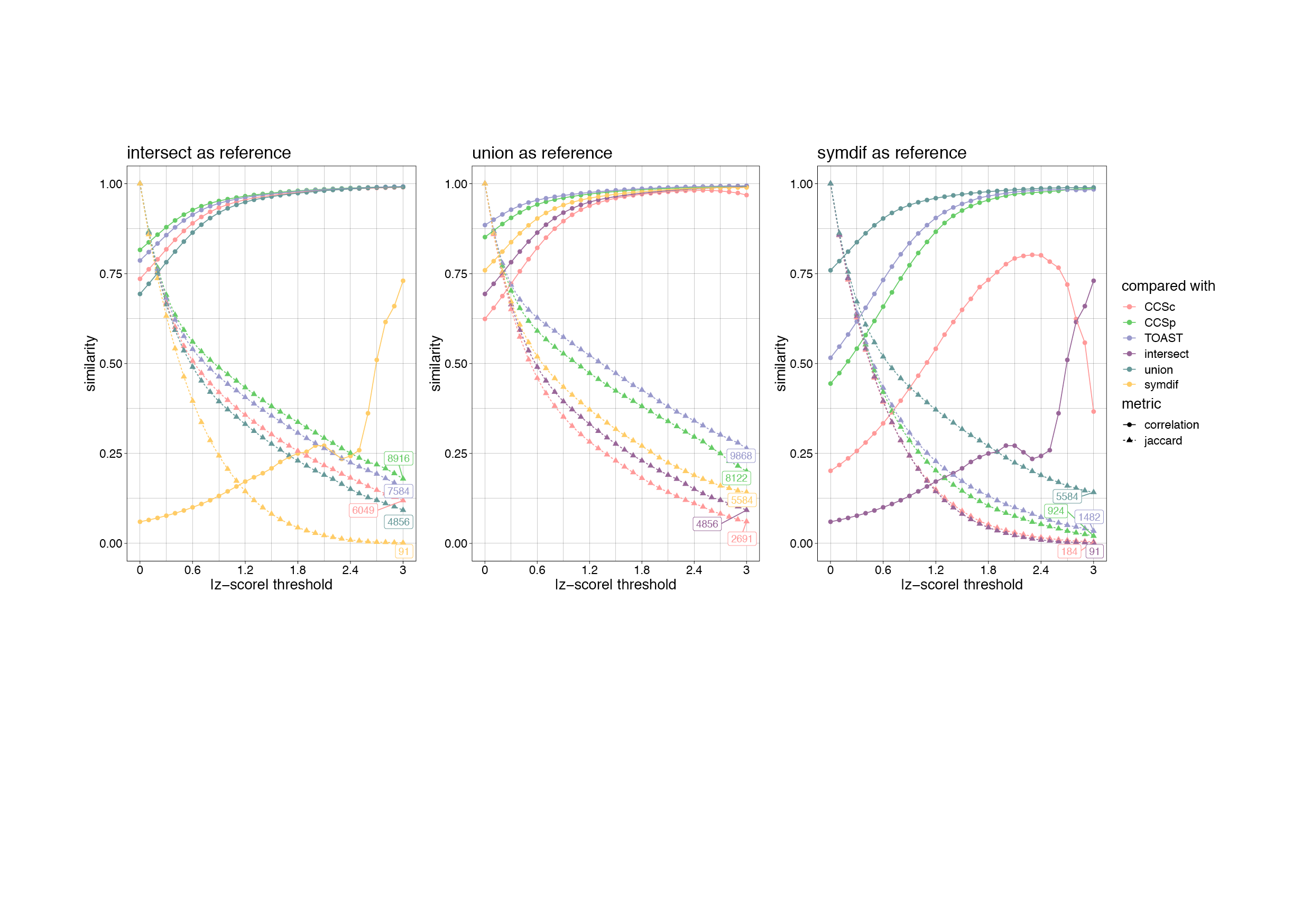


**results of overlap analysis.** Correlation is indicated with points, Jaccard index is indicated with triangles. (A) the other five phenotype definitions compared to intersect (B) the other five phenotype definitions compared to union (C) the other five phenotype definitions compared to symmetric difference

**Supplemental figure 4. Overall overlap analysis**

A small set of SNPs is shared between all phenotype definitions. To complement the pairwise overlap analyses, overall Jaccard index was calculated. Jaccard index is plotted on the y-axis, the absolute z-score threshold is plotted on the x-axis. The number of shared SNPs at z = 3 is indicated in the boxes.

**
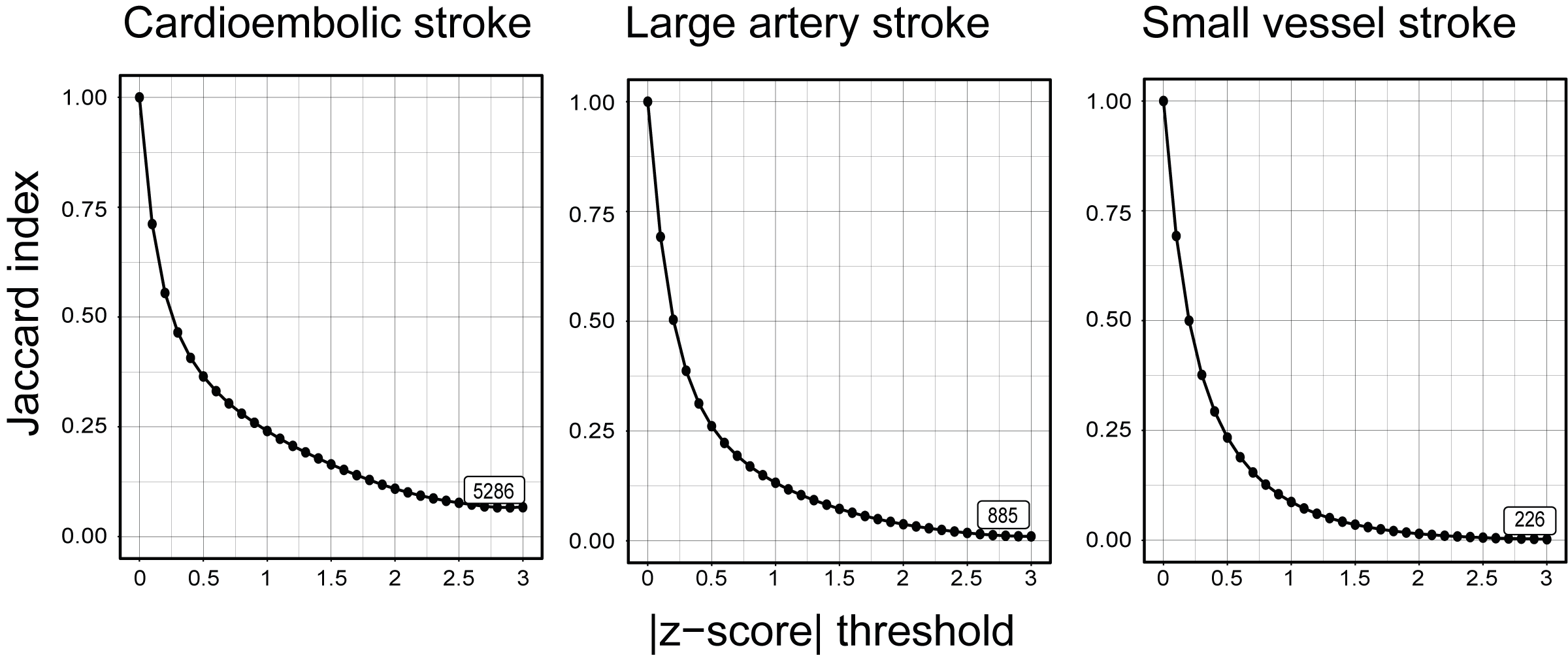
**

**Supplemental figure 5. Intersect most often shows the strongest effect**

For all previously known hits, odds ratios and their confidence intervals in each of the GWAS described here, are plotted. Colors indicate subtype, symbols indicate phenotype definition.

**See attached file FigS5.tiff**

**Supplemental figure 6. Regional association plots**

Regional association plots were made using Locuszoom ^[[1]](#footnote-1)^. The reference population used for LD calculation is 1000 genome phase 3 version 5, European.

rs11697087 rs11065979


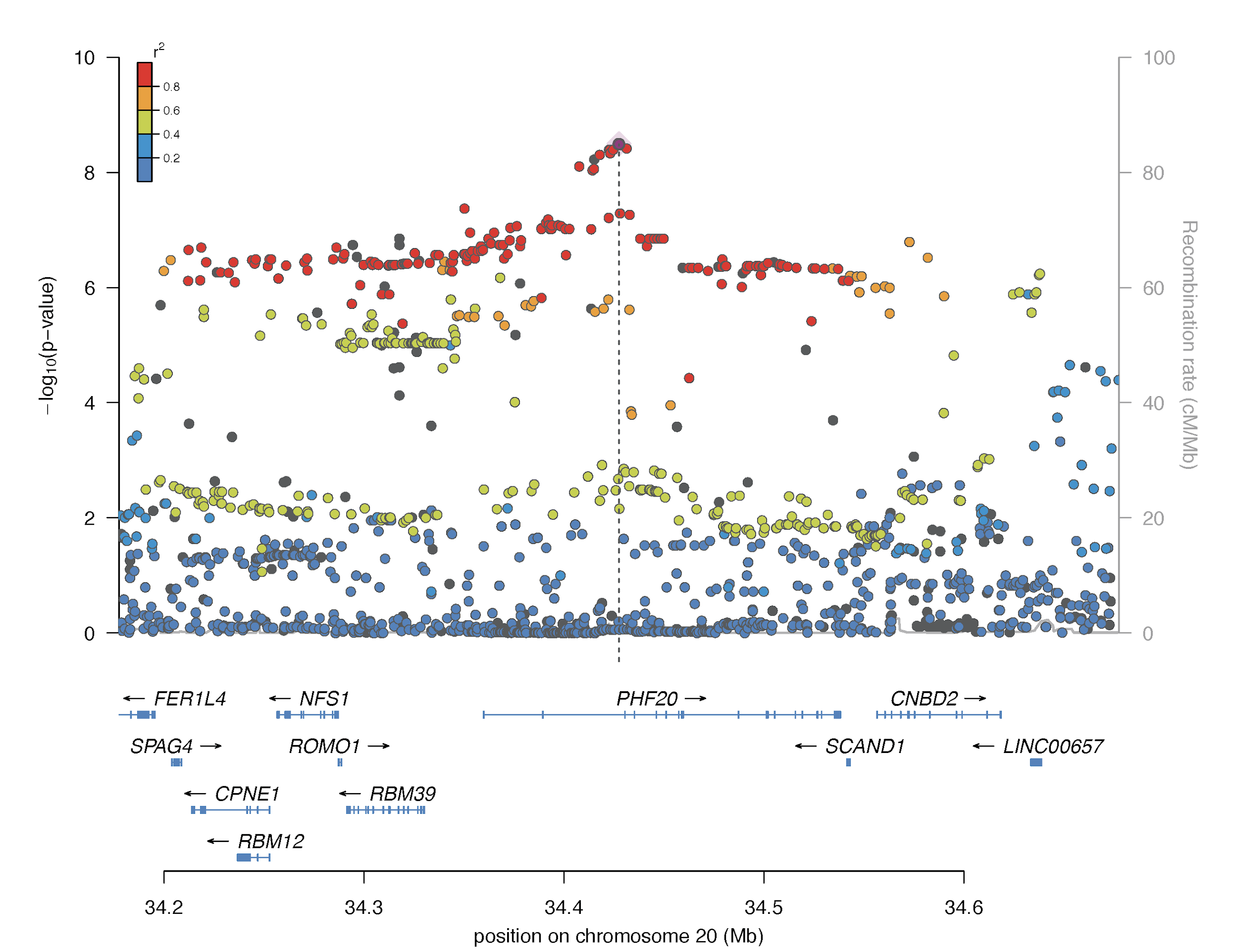
**
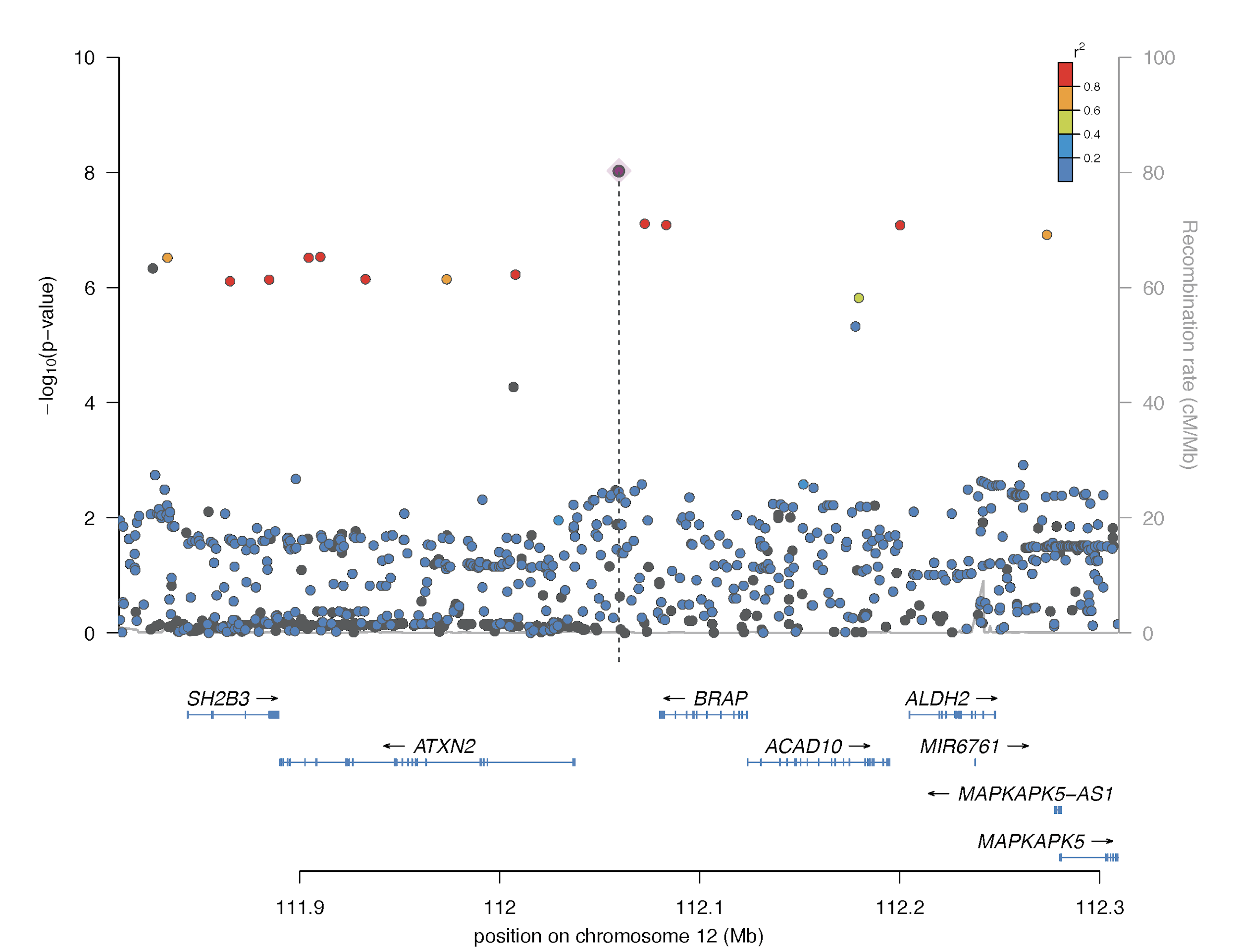
**

rs10029218 rs3790099

**
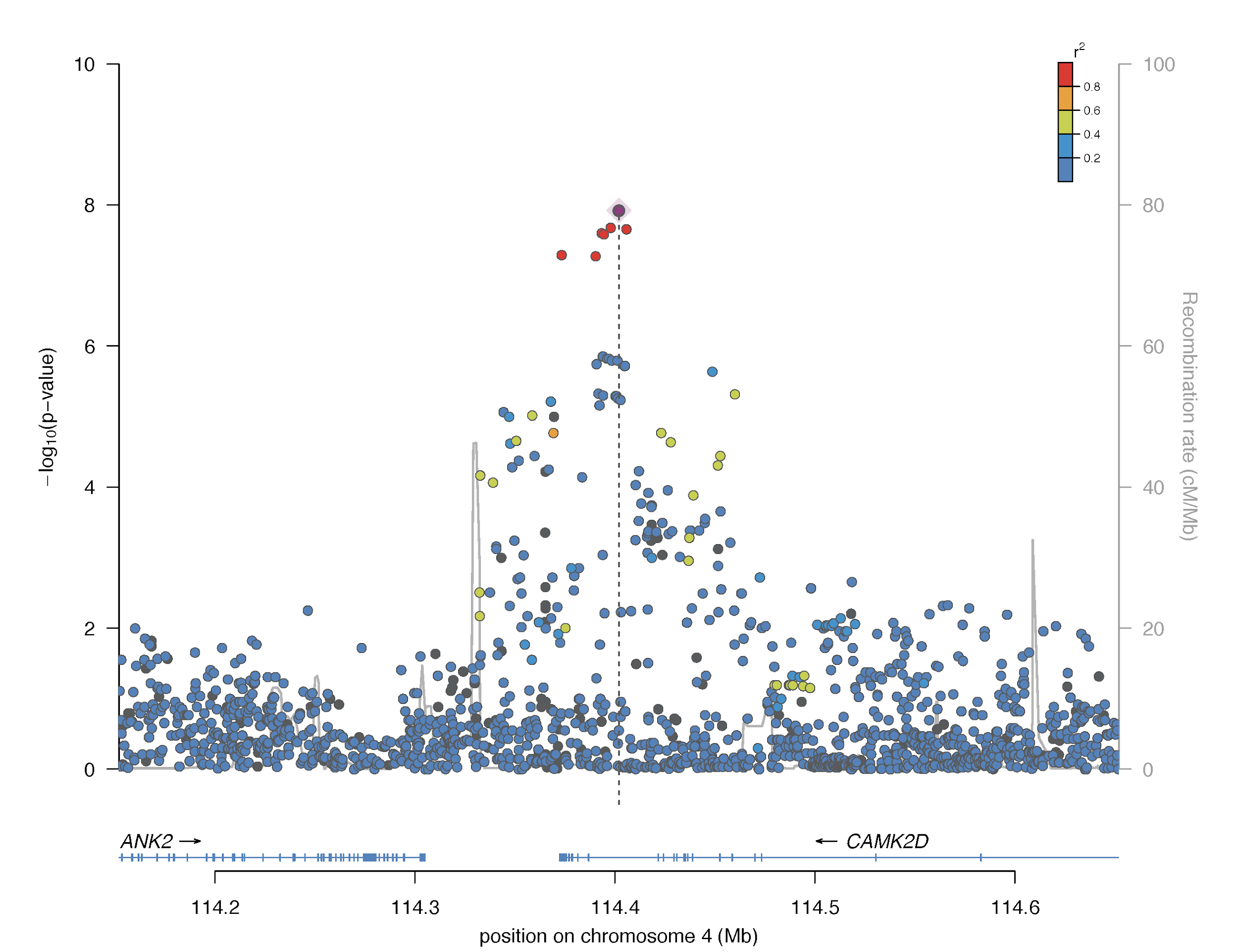

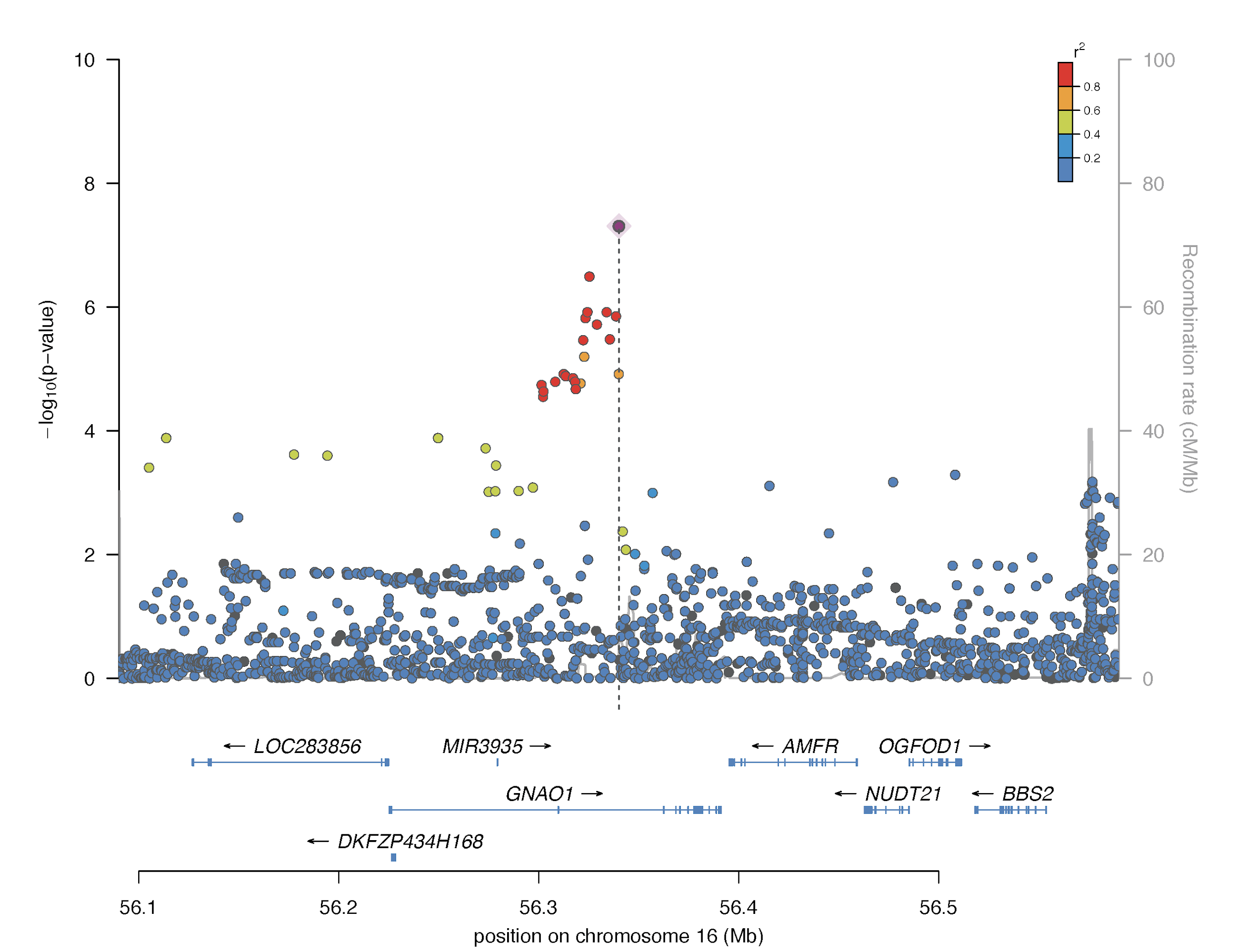
**rs2169955


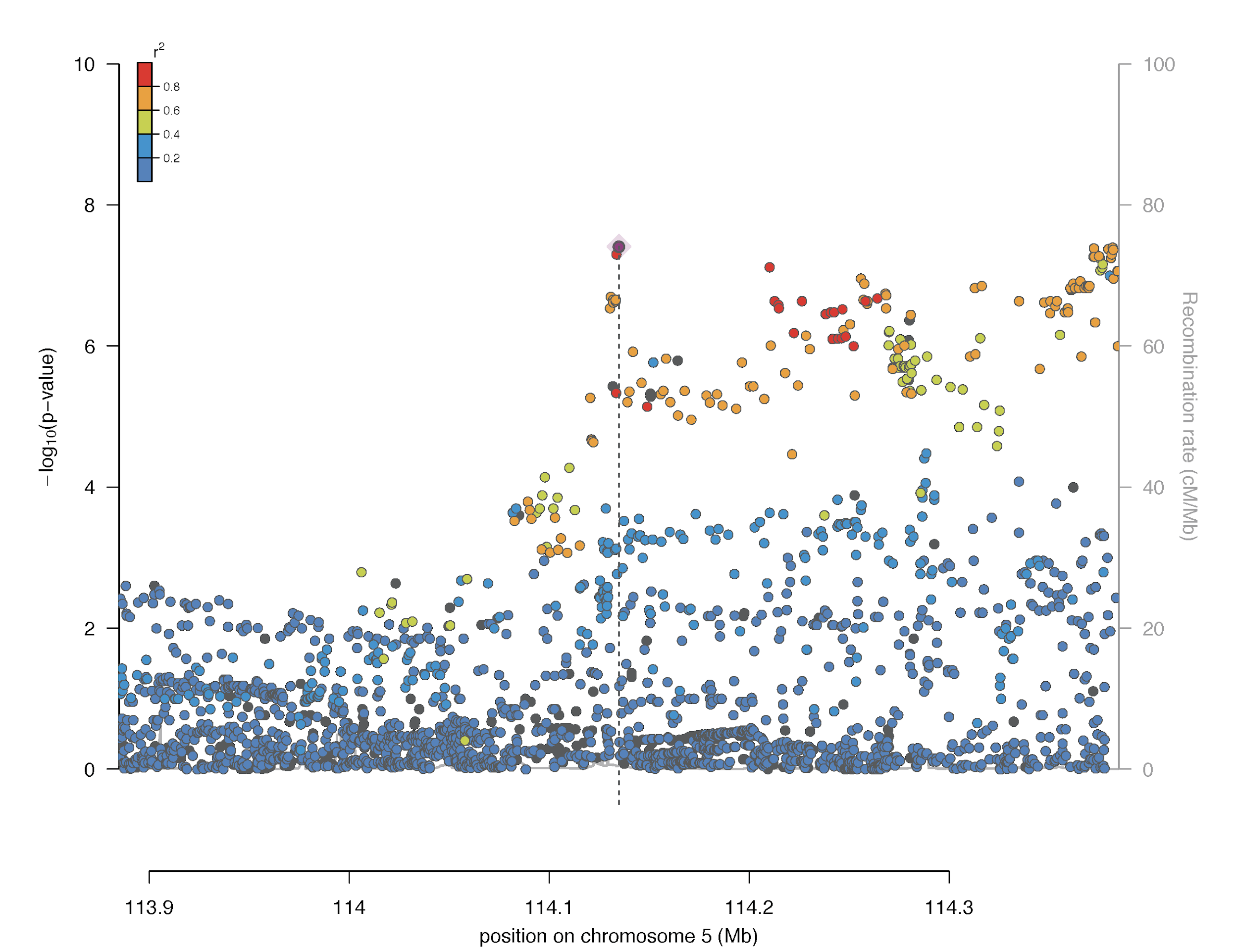


1. Pruim RJ, Welch RP, Sanna S, Teslovich TM, Chines PS, Gliedt TP, et al. LocusZoom: regional visualization of genome-wide association scan results. Bioinformatics. 2010;26: 2336–2337. [↑](#footnote-ref-1)
