# Supplementary figures and images for: Alternate approach to stroke phenotyping identifies a genetic risk locus for small vessel stroke
Source: Eur J Hum Genet. 2020 Feb 11;28(7):963–72. doi: 10.1038/s41431-020-0580-5 (PMC7316747; doi:10.1038/s41431-020-0580-5)

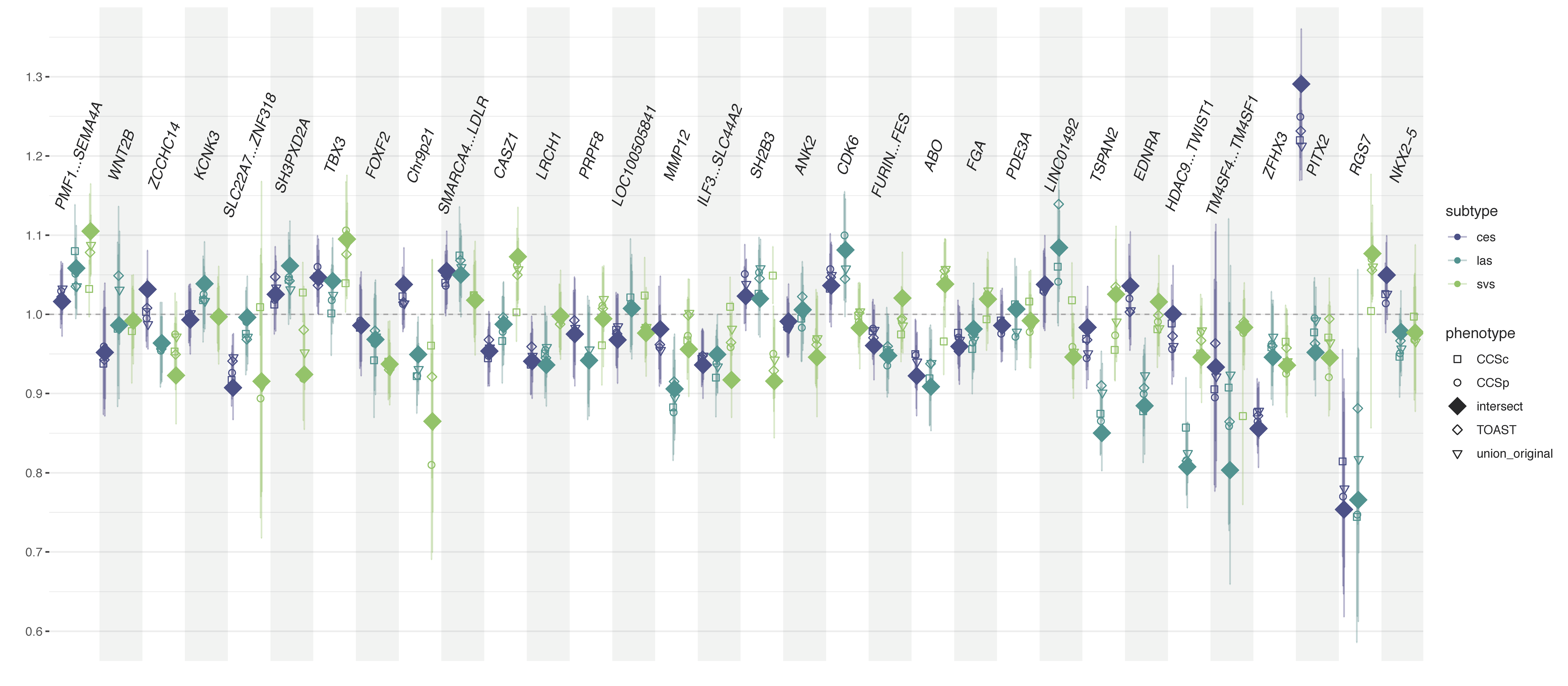

Supplement: Supplementary file 4 — Figure S5 [file 41431_2020_580_MOESM4_ESM.tif]
